# KG-DFI: A Prediction of Drug–Food Interactions Based on Knowledge Graph Embedding

**DOI:** 10.34133/csbj.0205

**Published:** 2026-08-18

**Authors:** Mingi Kang, Myoung Jin Lee, Sunyong Yoo

**Affiliations:** ^1^ Department of Intelligent Electronics and Computer Engineering, Chonnam National University, Gwangju, Republic of Korea.; ^2^ R&D Center, MATILO AI Inc., Gwangju, Republic of Korea.

## Abstract

Drug–food interactions (DFIs) can substantially affect patient safety and therapeutic outcomes. However, most existing computational approaches represent foods by decomposing each food into individual constituent molecules, which captures only limited aspects of foods and aligns poorly with real-world clinical practice, where dietary decisions are made at the level of whole foods rather than isolated components. This study constructed a knowledge graph centered on DFIs by integrating diverse biomedical databases covering food constituents, drug-related knowledge, and compound–gene interactions. Here, we propose a knowledge graph-based drug–food interaction (KG-DFI) prediction model that treats foods as complete entities rather than being decomposed into individual compounds. Notably, a KG-DFI model employs relational graph convolutional networks to learn food-entity representations that capture both structural and relational information from the knowledge graph. In addition, we incorporated a cross-attention mechanism that enables food entities to selectively attend to relevant drug characteristics, effectively capturing complex interaction patterns. In predicting DFIs, the KG-DFI model achieved an area under the receiver operating characteristic curve value of 0.9292 and an F1-score of 0.8640. Additionally, the KG-DFI model predicted the 4 specific interaction types (possible, positive, negative, and harmful) among observed DFIs, achieving an area under the receiver operating characteristic of 0.9163 and a weighted F1-score of 0.8801. This framework enables practical clinical decision support by predicting interactions using representations of food as entities, aligning with how healthcare providers deliver dietary recommendations to patients.

## Graphical Abstract

**Figure:**
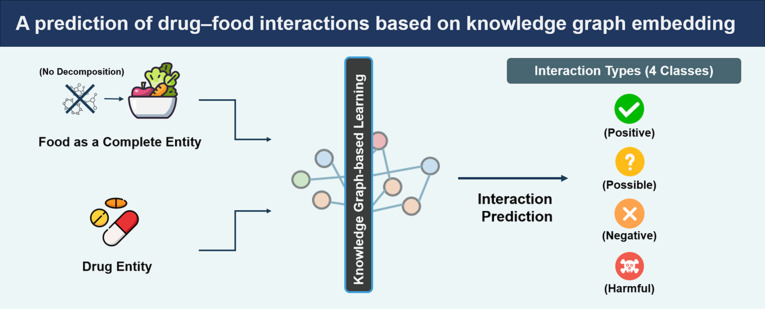

## Introduction

Medications are widely used for health maintenance and disease treatment, but dietary factors can markedly influence drug efficacy. Food intake represents another critical factor that can substantially affect therapeutic outcomes when drugs are coadministered with meals [1,2]. A drug–food interaction (DFI) refers to a situation in which food consumption alters drug pharmacokinetics, pharmacodynamics, or bioavailability, thereby affecting treatment outcomes by either enhancing or reducing drug effects or causing adverse effects [3]. Well-characterized examples include grapefruit juice, which dramatically enhances drug bioavailability through irreversible CYP3A4 inhibition, as exemplified by the withdrawal of the antihistamine terfenadine due to severe cardiac arrhythmias [4,5]. Moreover, polypharmacy affects a substantial proportion of patients, and dietary supplement use is increasing; thus, accurate DFI prediction is essential to ensure patient safety and therapeutic efficacy [6,7]. Additionally, recognizing and managing these interactions is crucial for minimizing patient harm and optimizing therapeutic outcomes [8].

Current knowledge of DFIs primarily comes from clinical research and biomedical literature that examine how food affects drug efficacy [1,9,10]. These studies have been limited to a small number of drugs and specific foods, focusing on revealing mechanisms from a biomedical perspective. These traditional approaches are constrained by cost, time demands, and ethical considerations and typically focus on well-characterized interactions, providing limited insight into broader experimental conditions [11]. Consequently, these constraints have motivated the exploration of computational alternatives.

Notably, the success of computational frameworks in bioinformatics has led to the subsequent application of these frameworks for predicting DFIs—several computational methods have been proposed for this purpose [12]. DeepDDI, originally developed for drug–drug interaction classification, was extended to predict interactions between drugs and food constituents [6]. DFinder employed network-based analysis to identify potential DFI, while FDmine utilized machine learning techniques with molecular descriptors [13,14]. Nonetheless, despite these technological advances, the application of computational techniques for predicting DFIs has been hampered by methodological limitations. Previous computational approaches have relied on component-level analysis, in which foods are decomposed into individual molecular constituents, and the associated interactions with drugs are predicted using structure-similarity profile derived from Simplified Molecular Input Line Entry System representations [13]. While these studies demonstrated the feasibility of computational DFI prediction, the component-level approach has critical limitations; for example, this approach introduces a fundamental misalignment with clinical practice, in which dietary decisions are made at the level of whole foods, such as “avoid grapefruit juice with this medication” [1]. Additionally, this approach fails to capture the complex synergistic and antagonistic effects present within food matrices [15]. Structure-similarity profile-based molecular similarity metrics also suffer from the activity cliff problem, in which structurally similar compounds can exhibit different biological activities [16]. Although recent developments, such as the Diet–Drug Interactions Database (DDID), have yielded datasets containing 23,950 interaction records and helped address data scarcity issues, the key challenge of predicting interactions directly from food, necessary for clinical application, remains unresolved [17].

To address these limitations and represent food as an independent entity within computational frameworks, knowledge graph approaches offer a promising alternative. Graphs composed of entities and the associated interconnected relationships provide a natural representation of complex biomedical systems [18]. Moreover, knowledge graph embedding techniques have demonstrated exceptional capabilities for integrating heterogeneous biomedical data types and for discovering novel relationships between biological entities [19,20]. Meanwhile, graph neural networks that aggregate information from entity neighborhoods have shown superior performance in biomedical prediction tasks compared to traditional approaches [21,22]. Recent advances in graph neural network architectures for biomedical prediction—including curvature-enhanced graph convolution, knowledge graph-based neural networks, multiview feature encoding, and automated knowledge graph construction—have further established heterogeneous graph representations as the leading paradigm for biomedical interaction modeling [23–28]. However, to our knowledge, no prior computational framework has treated individual foods as whole-food entities within a knowledge graph for direct DFI prediction at the food level, which constitutes a critical research gap.

Thus, this work presents a computational framework for predicting DFIs at the whole-food entity level using knowledge graph embedding, to our knowledge the first to predict DFIs directly from food entities rather than decomposing foods into individual molecular constituents [29].

The contributions of this work include: (a) a knowledge graph embedding framework that, to our knowledge, is the first to predict DFIs at the whole-food entity level using knowledge graph embedding, directly addressing the fundamental misalignment between molecular-level computation and food-level clinical guidance; (b) proposing a neural network architecture that combines relational graph convolutional networks (R-GCNs) with cross-attention mechanisms to capture both compositional information and relational context; (c) conducting extensive evaluations that demonstrate improved prediction of interactions for commonly consumed foods while maintaining high accuracy across diverse drug classes. The source code and installation guidelines for knowledge graph-based drug–food interaction (KG-DFI) are openly available at https://github.com/bmil-jnu/KG-DFI.

## Materials

### Data sources and preprocessing

This study integrated 3 databases to construct a comprehensive knowledge graph for DFI prediction: FooDB, the Drug Repurposing Knowledge Graph (DRKG), and the Comparative Toxicogenomics Database (CTD) [30–33]. Each database was systematically curated to ensure data quality and integration compatibility.

FooDB provided food composition data, including information on dietary foods and the associated constituent compounds. This study focused exclusively on the “foods” category, excluding “herbs”, as herbs are typically administered as pharmacological agents rather than consumed as dietary staples, and their inclusion would conflate DFIs with herbal–drug effects. After filtering, 98,603 unique food–compound associations remained, comprising 894 food items and 11,580 unique component compounds. Beyond this conceptual distinction, excluding herbs also reflects a practical data-consistency concern, as herb–drug interactions are curated through separate pharmacovigilance resources that apply different dosing and annotation standards; incorporating herbs without a DDID-consistent herb dataset would therefore risk label inconsistency rather than a coherent training signal. Extending KG-DFI to herbs remains a promising direction for future work.

DRKG is a comprehensive biomedical knowledge graph that integrates biomedical information from 6 databases, including DrugBank, Hetionet, GNBR, STRING, IntAct, and DGIdb, along with literature evidence from COVID-19 studies. A drug subgraph was subsequently constructed by retrieving all biological entities and relationships directly connected to compound entities, yielding 1,264,354 triplets with 4,687 compound entities encompassing interactions with genes, diseases, side effects, anatomical therapeutic chemical, and pharmacological classes.

The CTD provided mechanistic chemical–gene interaction data. Only compounds with Chemical Abstracts Service registry numbers were retained and mapped to standardized database identifiers, including PubChem CID, ChEMBL, and DrugBank, to enable integration with FooDB and DRKG. After filtering and deduplication, 422,276 unique compound–gene interaction triplets spanning 21 mechanistic interaction types were obtained for subsequent connectivity-based integration. Although Chemical Abstracts Service-number-based filtering may underrepresent less-characterized compounds lacking this identifier, this constraint ensures standardized cross-database integration and is consistent with established multidatabase knowledge graph construction practices. Detailed curation and identifier-mapping procedures are described in File S1.

### Drug–food knowledge graph

We constructed a knowledge graph centered on drug–food relationships by integrating curated data from FooDB, DRKG, and CTD. FooDB and DRKG were first combined through shared compound entities using standardized chemical identifiers. The integration of FooDB and DRKG yielded a baseline knowledge graph of 1,362,957 triplets.

To enrich mechanistic information while maintaining graph connectivity, CTD triplets were integrated by retaining only those in which both the compound and gene entities were present in the baseline knowledge graph, yielding 85,010 triplets from the original 422,276 curated interactions. This connectivity-based approach covered 429 compounds and 7,468 genes with validated mechanistic interaction data, ensuring that all newly added relationships could be contextualized within the established biological network while preserving the overall graph structure. Because this criterion retains only entities already shared across multiple source databases, it likely favors well-characterized genes and pathways (e.g., CYP450 enzymes), potentially limiting coverage of interactions mediated by less-studied biological mechanisms.

The final customized knowledge graph contains 41,698 entities distributed across 7 categories with 71 relation types (Fig. S1). Notably, to prevent data leakage, we completely removed direct DFI information from the background knowledge graph. Graph connectivity analysis confirmed that all entities remained interconnected, with no isolated nodes in the final network.

After preprocessing and integration, the curated databases comprised 98,603 triplets from FooDB, 1,264,354 from DRKG, and 85,010 from the CTD, resulting in a final customized knowledge graph of 1,447,967 triplets.

### Data sources for model training

We collected DFI data for model training from the DDID [17]. The DDID is a manually curated public database dedicated to DFI research. Moreover, the DDID provides basic information on drugs, foods, and herbs, as well as the effects of interactions. We obtained 1,642 validated DFIs, which were categorized into 4 interaction types (Table 1). The dataset exhibited severe class imbalance, with possible interactions comprising 73.7%, followed by positive interactions at 18.8%, negative interactions at 5.1%, and harmful interactions at 2.4%. During preprocessing, we retained only drug–food pairs in which both entities existed in our constructed knowledge graph to ensure that all interactions could be contextualized within the network. For binary classification tasks, we generated 1,642 negative samples by randomly sampling noninteracting drug–food pairs, resulting in a balanced training dataset of 3,284 samples. Binary interaction labels were assigned based solely on whether a drug–food pair was recorded in DDID (presence in DDID = interacting; absence = noninteracting), with no dose or concentration thresholds applied, consistent with the curated annotation scheme of the source database.

**Table 1.** Data per DFI relationship in the DDID dataset

	Possible	Positive	Negative	Harmful	Total
DDID dataset	1,209 (73.7%)	309 (18.8%)	84 (5.1%)	40 (2.4%)	1642

DFI, drug–food interaction; DDID, Diet–Drug Interactions Database

### External dataset for validation

We used DrugBank (version 5.1.5) as an external dataset to evaluate the generalizability and clinical relevance of our predictive model [34]. To construct the validation reference dataset, we applied a 3-step filtering procedure to extract oral drugs from DrugBank that had been approved by the United States Food and Drug Administration. This procedure yielded 986 unique Food and Drug Administration-approved oral drugs, corresponding to approximately 5.8% of all DrugBank compounds. These entries were subsequently mapped to a customized knowledge graph containing 41,698 nodes, yielding 925 drugs with existing DrugBank IDs and 889 food entities (after excluding broad category-level entries such as fruits and beverages), for a total of 822,325 DFI pairs. The step-by-step filtering criteria and intermediate counts are provided in File S2. The 822,325 DrugBank-derived drug–food pairs represent all possible combinations of the 925 DrugBank-mapped drug entities and the 889 food entities retained after excluding broad category-level entries such as fruits and beverages. Because the DDID interactions used for model training, validation, and testing are themselves drawn from this same entity pool, a substantial fraction of DDID pairs is necessarily contained within this combinatorial space (957 of the 1,642 positive DDID pairs, 58.3%); this reflects the shared entity space from which both sets are drawn rather than a design flaw in the external validation. The “Case study: Validation of predicted drug–food interactions” section describes how the specific top-ranked predictions selected for case study analysis were individually confirmed to be absent from the DDID data used for training, validation, and testing.

## Methods

### Overview and problem formulation

**Fig. 1.**
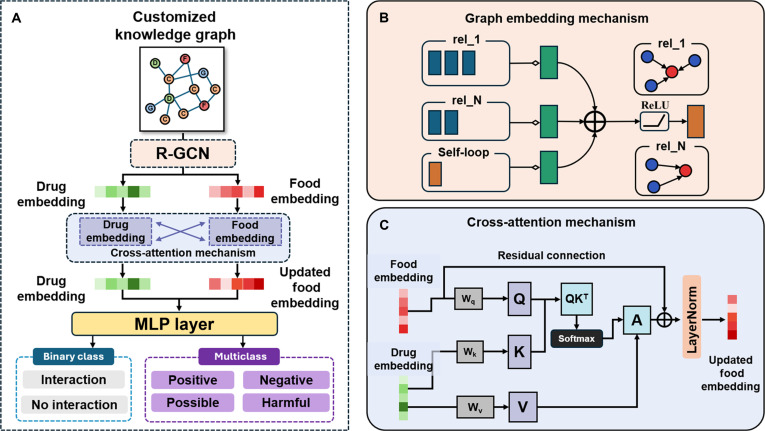
Architecture of the proposed knowledge graph-based drug–food interaction (KG-DFI) model for DFI predictions. (A) Overall pipeline of the KG-DFI framework. The biomedical knowledge graph is encoded using an relational graph convolutional network (R-GCN) to generate drug and food embeddings, which are then updated via a cross-attention mechanism. The updated food embedding and the original drug embedding are concatenated and passed through multilayer perceptron (MLP layers to generate predictions, supporting both binary classification (interaction/no interaction) and multiclass prediction (positive/possible/negative/harmful). (B) Graph embedding mechanism of the R-GCN encoder, which aggregates entity representations across multiple relation types and a self-loop connection to produce node embeddings. (C) Cross-attention mechanism, where the food embedding serves as the Query and the drug embedding provides the Key and Value. The resulting attention matrix is applied to the Value to produce an updated food embedding that captures the influence of drug characteristics on food-related representations.

This study developed a KG-DFI prediction model, a knowledge graph-enhanced framework for predicting DFIs. Given the food entity f and the drug entity d from our customized knowledge graph, the model can predict the probability of a clinically meaningful interaction between each entity (Fig. 1).

### Task definition

We formulated the DFI predictions as 2 tasks. First, we addressed the binary classification to determine whether the drug–food pairs $\left(f_{i},d_{j}\right)$ exhibit clinically meaningful interactions (positive: 1) or not (negative: 0). For each food entity $f_{i}$ and drug entity $d_{j}$, the system outputs interaction likelihood as probability scores $\;p_{\mathrm{ij}}\in \left[0,1\right]$. Second, we extended this to the multiclass prediction to characterize interaction types more precisely. Interactions are categorized into “positive”, “negative”, “harmful”, and “possible” classes, and models output interaction scores for each.

### Knowledge graph encoder

Entity embeddings are learned using the R-GCN to handle our heterogeneous biomedical graph with 71 distinct relation types (Fig. S2) [35]. Unlike standard GCNs that treat all edges equally, the R-GCN assigns relation-specific weight matrices to different edge types, enabling the model to distinguish between drug–target interactions, food–compound containment, and other biomedical relationships.

The node update rule for R-GCN is formulated as:

$$h_{i}^{\left(l+1\right)}=\sigma \left(W_{0}^{\left(l\right)}\;h_{i}^{\left(l\right)}+\sum_{r\in R}\sum_{j\in N_{i}^{r}}\frac{1}{\left|c_{i,r}\right|}\;W_{r}^{\left(l\right)}\;h_{j}^{\left(l\right)}\right)$$ (1)

where $h_{i}^{\left(1\right)}$ denotes the hidden state of the entityi at layer l, $N_{i}^{r}$ represents neighbors of the entity i connected via relation r, $W_{r}^{\left(l\right)}$ is the relation-specific transformation matrix, and $c_{i,r}$ is a normalization constant. Each R-GCN layer assigns an independent weight matrix to each of the 73 relation types (71 semantic relation types plus self-loop and auxiliary relations), following the R-GCN formulation [35]. Our architecture uses 3 R-GCN layers with 256-dimensional embeddings, selected through a systematic grid search of 144 configurations spanning embedding dimension (64, 128, 256), number of R-GCN layers (1 to 4), focal loss gamma (0.5, 1.0, 2.0, 3.0), and cross-attention heads (2, 4, 8) (File S5), enhanced by layer normalization and residual connections.

### Cross-attention mechanism

DFIs are modeled through multihead cross-attention adapted from Transformer architectures [36]. This mechanism enables food entities to selectively attend to relevant drug characteristics, identifying which drug properties are most important for specific food entities. Similar cross-attention strategies have demonstrated effectiveness in biomedical interaction prediction, supporting the validity of this modeling approach [37,38].

The attention mechanism operates with 4 attention heads, where food embeddings serve as queries while drug embeddings act as keys and values:

$$\text{Attention}\left(Q,K,V\right)=\text{softmax}\left(\frac{QK^{T}}{\sqrt{d_{k}}}\right)V$$ (2)

where Q represents query vectors (food embeddings), K,V are key and value vectors (drug embeddings), and $d_{k}$ is the dimension per attention head. The attention output is combined with original food embeddings via residual connections and layer normalization.

### Model training

For model training, positive samples comprised 1,642 validated DFI pairs as described in Materials. Negative samples were generated via stratified random sampling of noninteracting drug–food pairs, maintaining a 1:1 ratio to ensure a balanced distribution of the training dataset. The combined dataset was split into training (70%), validation (15%), and test (15%) sets using stratified random sampling (random state = 42) to ensure consistent class distribution and reproducibility across experiments.

The concatenated attention-enhanced food and drug embeddings were processed through a multilayer perceptron (MLP). The final sigmoid layer produces interaction probabilities for binary classification.

The MLP predictor comprises 4 fully connected layers (dimensions 512→128→128→1) with ReLU activations, batch normalization, and dropout (*P* = 0.3) applied between layers to mitigate overfitting. The full model was trained with the Adam optimizer (learning rate = 5 × $10^{-4}$), batch size 64, and early stopping with a patience of 15 epochs based on validation-set performance. Full architectural and training details, together with formal definitions of the evaluation metrics, are provided in File S3.

To address class imbalance in DFI prediction, we employed focal loss [39]:

$$\mathrm{FL}\left(P_{t}\right)=-\alpha_{t}{\left(1-p_{t}\right)}^{\gamma }\text{log}\left(p_{t}\right)$$ (3)

where $P_{t}$ is the predicted probability for the true class, $\alpha_{t}$ controls class weighting, and γ down-weights easy examples to focus learning on hard cases. For the binary task, negative samples are drawn to yield a balanced 1:1 ratio (“Model training” section, first paragraph), so α was held fixed at 0.25, the standard default from the original formulation [33], while *γ* = 2.0. For the multiclass task, which exhibits severe class imbalance (Possible: 73.7%, Harmful: 2.4%, Negative: 5.1%), α was instead set to a per-class weight vector computed via balanced class weighting, and *γ* = 1.0 was selected via the systematic grid search described in the “Knowledge graph encoder” section (File S5). We prioritized *γ* for tuning in the multiclass task because it directly controls the degree of down-weighting applied to easy examples, which has the most direct effect on class-imbalance handling in that setting.

We note that, over the course of training, validation-set weighted F1 improved even as validation-set focal loss increased. This pattern is expected under class-imbalance-aware objectives: Because per-class weighting assigns a substantially larger penalty to errors on the rare Harmful and Negative classes than to errors on the abundant Possible class, a small number of low-confidence or incorrect predictions on these rare classes can inflate the aggregate loss even while the argmax-based decision boundary—and therefore weighted F1—continues to improve. Model selection was accordingly based on validation-set weighted F1 rather than validation loss, consistent with standard practice for class-imbalanced classification.

Models were trained with the Adam optimizer and early stopping. Detailed MLP architecture, hyperparameters, and training configuration are provided in File S3. Hyperparameters for traditional baseline classifiers (support vector machine [SVM], random forest [RF], XGBoost, and LightGBM) were selected via 5-fold cross-validation grid search on the training set; the search grids and optimal configurations are provided in File S5.

Model performance was assessed using comprehensive metrics for both binary and multiclass classification tasks. Standard classification metrics include accuracy, precision, recall, F1-score, and Cohen’s kappa. Additionally, the area under the receiver operating characteristic (AUROC) curve evaluates the discriminative ability of the model across all classification thresholds. In contrast, the area under the precision–recall (AUPR) curve assesses performance and is particularly suited to the imbalanced datasets common in biomedical applications. Formal definitions of all evaluation metrics are provided in File S3.

## Results

### Overall experimental results

We conducted a comprehensive evaluation of the proposed KG-DFI framework on 2 prediction tasks: binary DFI detection and multiclass interaction classification. Our experiments compared the performance of the KG-DFI prediction model against multiple baseline approaches to assess the predictive accuracy of the proposed method.

The evaluation encompassed traditional machine learning models, including SVM, RF, XGBoost, and LightGBM, each paired with its own independently trained R-GCN knowledge graph encoder, rather than sharing the encoder trained for KG-DFI, so that performance differences reflect classifier capability rather than any representational advantage inherited from the KG-DFI training procedure, as well as a baseline R-GCN model without cross-attention mechanisms [40–43]. This experimental design enables direct assessment of the architectural contributions of our proposed cross-attention module while maintaining consistent knowledge representation across all models. Specifically, each traditional classifier (SVM, RF, XGBoost, and LightGBM) received as input the concatenated entity embedding pairs produced by an independent R-GCN encoder trained under conditions identical to those used in KG-DFI, ensuring that any performance differences reflect architectural rather than representational advantages.

### Binary classification results

Table 2 presents the comparative performance of all models on the binary DFI prediction task. Our proposed KG-DFI model achieved the best performance across all evaluation metrics, with an accuracy of 0.8632, F1-score of 0.8640, AUROC of 0.9292, and AUPR of 0.9192. Among the baseline methods, XGBoost achieved strong performance with an F1-score of 0.8485.

**Table 2.** Performance comparison of different models on the DFI prediction task (binary class). Values in boldface indicate the best performance for each metric.

Model	Accuracy	F1-score	AUROC	AUPR
KG embedding + SVM	0.7994	0.7925	0.8569	0.8374
KG embedding + RF	0.8298	0.8323	0.9013	0.8959
KG embedding + XGBoost	0.8480	0.8485	0.9097	0.8922
KG embedding + LightGBM	0.8450	0.8459	0.9129	0.8928
KG-DFI without cross-attention	0.7660	0.7631	0.8300	0.7859
KG-DFI (ours)	**0.8632**	**0.8640**	**0.9292**	**0.9192**

AUROC, area under the receiver operating characteristic; SVM, support vector machine; RF, random forest; KG, knowledge graph; DFI, drug–food interaction

The full KG-DFI model outperformed KG-DFI without cross-attention, improving F1-score from 0.7631 to 0.8640 and AUROC from 0.8300 to 0.9292, representing improvements of 10.09 and 9.92 percentage points, respectively. This demonstrates the effectiveness of the cross-attention mechanism in capturing DFI patterns. While knowledge graph embeddings from R-GCN provide a foundation, the cross-attention module is essential for leveraging heterogeneous biomedical information to predict the DFIs. Additional comparisons against a graph attention network (GAT)-based encoder variant, together with several knowledge graph embedding and heterogeneous graph neural network baselines, are reported in File S7.

### Multiclass classification result

Table 3 presents the performance comparison for multiclass DFI predictions. The test set exhibits class imbalance in sample counts: harmful (2.4%), positive (19.1%), possible (73.9%), and negative (4.9%). All metrics were calculated using weighted averaging to reflect this distribution. The KG-DFI model achieved the highest performance with an AUROC of 0.9163, an AUPR of 0.9234, and a weighted F1-score of 0.8801. Cohen’s kappa, which measures agreement beyond chance by correcting for random agreement expected from class distribution, indicated substantial agreement (0.6646), suggesting that performance improves due to increased discriminative ability rather than from exploiting class imbalance. This represents a 17.0% improvement over the best baseline with a Cohen’s kappa value of 0.5682. To quantify the statistical reliability of KG-DFI, we estimated 95% confidence intervals (CIs) via 1,000-iteration bootstrap resampling on the test set: F1-score = 0.8801 (95% CI: 0.8368 to 0.9163), AUROC = 0.9163 (95% CI: 0.8764 to 0.9496), AUPRC = 0.9234 (95% CI: 0.8912 to 0.9553), and Cohen’s kappa = 0.6646 (95% CI: 0.5621 to 0.7591). These narrow CIs confirm the stability and reproducibility of the reported performance. The harmful class, which constitutes 2.4% of the test set (6 of 247 samples), showed a recall of 0.50 (3 of 6 correctly identified); the 3 missed cases were misclassified as Possible (1) or Positive (2), and none were misclassified as Negative. A full sensitivity analysis for the harmful class is provided in File S4. Model calibration was assessed via the Expected Calibration Error and a reliability diagram (File S10); the model was found to be systematically overconfident, particularly for the rare Negative and Harmful classes.

**Table 3.** Performance comparison of different models on the DFI prediction task (multiclass). Values in boldface indicate the best performance for each metric.

Model	F1-score ^a^	AUROC ^a^	AUPR ^a^	Kappa
KG embedding + SVM	0.8194	0.8743	0.8612	0.5682
KG embedding + RF	0.8062	0.8874	0.8762	0.5299
KG embedding + XGBoost	0.8035	0.8729	0.8670	0.5242
KG embedding + LightGBM	0.7984	0.8515	0.8485	0.5164
KG-DFI without cross-attention	0.1499	0.8298	0.8415	0.1166
KG-DFI (ours)	**0.8801**	**0.9163**	**0.9234**	**0.6646**

AUROC, area under the receiver operating characteristic; KG, knowledge graph; DFI, drug–food interaction; AUPR, area under the precision–recall; SVM, support vector machine; RF, random forest

^a^ Weighted average.

The model without cross-attention performed poorly. The difference between precision and recall indicates a prediction bias toward the majority class, with high precision on dominant classes while failing to identify minority interactions. This contrast demonstrates the essential role of cross-attention in maintaining balanced predictions in the presence of class imbalance. Detailed per-class precision, recall, and F1-score values along with confusion matrices are provided in File S4.

Among the traditional machine learning approaches, SVM achieved the strongest baseline performance, with an F1-score of 0.8194 and a kappa of 0.5682, followed by RF, with an F1-score of 0.8062 and a Cohen’s kappa value of 0.5299. However, end-to-end training with the KG-DFI model yielded superior performance across all metrics, with balanced precision and recall confirming effective identification of both majority and minority interaction classes.

To assess how much of this performance depends on the compound–gene mechanistic edges contributed by CTD, we additionally compared the full knowledge graph against a reduced configuration in which these edges were removed; the results are reported in Table S4 (File S6).

### Case study: Validation of predicted drug–food interactions

To evaluate the accuracy and real-world relevance of our predictive framework, we conducted case studies using DFI pairs that received high model scores. Each top-scoring pair was systematically validated by cross-referencing against PubMed biomedical literature and clinical pharmacology resources to verify the predictions and assess the associated biological basis and supporting evidence in the literature (Table 4). We additionally confirmed that none of the top-ranked case study predictions presented in Table 4 correspond to drug–food pairs present in the DDID data used for model training, validation, or testing, ensuring that these specific high-scoring predictions reflect genuine generalization rather than memorization of previously observed interactions.

**Table 4.** Results of literature verification by predicted DFIs

Rank	Drug name	Food name	Predicted	Reported	Score	PubMed ID
1	Ferric maltol	Grapefruit	Positive	Negative	0.9984	6940487
2	Nicotinamide	Grapefruit	Negative	-	0.9954	No proof
3	Nicotinamide	Alcoholic beverages	Negative	Possible	0.9952	9349848
4	Ferric maltol	Mung bean	Positive	Negative	0.9947	20861210
5	Ferric maltol	Bilberry	Positive	Negative	0.9936	6940487
						35320401
						2507689
6	Etoposide	Grapefruit	Negative	Negative	0.9934	12389073
7	Nicotinamide	Pulses	Negative	-	0.9932	No proof
8	Bictegravir	Grapefruit	Positive	Positive	0.9926	40518723
						11737987
9	Zolpidem	Alcoholic beverages	Negative	Negative	0.9924	20458214
10	Metronidazole	Alcoholic beverages	Possible	Harmful	0.9921	31598109
11	Iohexol	Grapefruit	Positive	-	0.9919	No proof
12	Levodopa	Alcoholic beverages	Negative	-	0.9919	No proof
13	Iron sucrose	Grapefruit	Positive	Positive	0.9918	6940487
14	Ferrous fumarate	Grapefruit	Positive	Positive	0.9915	6940487
						35320401
						2507689
15	Ferric maltol	Oxheart cabbage	Positive	Possible	0.9915	6625831
						6940487
						2507689
16	Sodium bicarbonate	Alcoholic beverages	Possible	Possible	0.9914	38732226
17	Ferric maltol	Silver linden	Positive	Negative	0.9913	35947287
						25065123
						18211058
18	Gefitinib	Grapefruit	Positive	Positive	0.9913	17575239
19	Momelotinib	Milk (cow)	Negative	-	0.9912	No proof
20	Upadacitinib	Grapefruit	Positive	Positive	0.9911	31867699

DFI, drug–food interaction

Most high-score predictions involving grapefruit were substantiated by established evidence. Bictegravir and upadacitinib are metabolized by CYP3A4, and grapefruit—a strong CYP3A4 inhibitor—can increase drug exposure and adverse event risk [44–46]. Etoposide uniquely demonstrates reduced oral bioavailability when coadministered with grapefruit juice, in contrast to the typical pattern of CYP3A4 inhibition, underscoring the complex and variable effects of food constituents on drug absorption [47]. This directionally atypical prediction is consistent with the pharmacological literature: Etoposide is a well-established P-glycoprotein substrate, and grapefruit constituents are known to modulate transporter activity in addition to inhibiting CYP3A4, so the net effect on oral bioavailability depends on which pathway dominates. That KG-DFI recovers this nonstandard direction rather than defaulting to the more common CYP3A4-inhibition-increases-exposure trend suggests that the model’s relational message passing integrates signal from multiple mechanistic pathways rather than relying on a single dominant biological cue. Gefitinib shows increased systemic exposure due to CYP3A4 inhibition by grapefruit [48].

Iron-based drugs showed positive interactions when coadministered with vitamin C-rich foods, leading to improved absorption and hematological status [49–51]. Polyphenol- and flavonoid-rich foods, such as those containing quercetin and kaempferol, abundant in silver linden, have been shown to chelate and reduce the bioavailability of iron, thereby inhibiting iron absorption [52–55]. Ferric maltol, when combined with mung bean supplementation, produced a marked improvement in iron bioavailability in vivo, supporting a synergistic effect on iron repletion [56].

Alcohol–drug interactions were also predicted and confirmed by established evidence. Zolpidem combined with alcohol results in enhanced sedative effects and increased risk of central nervous system depression [57]. Coadministration of metronidazole and alcohol is well documented to provoke disulfiram-like reactions [58]. Sodium bicarbonate, when used alongside alcohol, was shown to decrease alcohol consumption in animal studies, potentially by altering systemic pH or reward pathways [59]. Additionally, nicotinamide with alcohol alleviated ethanol-induced hepatic toxicity in human studies [60]. It should be noted that the predictions in Table 4 are based on qualitative interaction labels and do not incorporate dose information; clinical relevance may vary depending on the quantities of food consumed and specific drug dosage regimens. Directional misclassification patterns observed among the Table 4 predictions, together with graph-traversal analyses of the multihop pharmacological pathways underlying these predictions, are examined in Discussion, with full connectivity comparisons and path enumerations reported in Files S8 and S9.

## Discussion

This study proposes a KG-DFI prediction model, a knowledge graph-based framework that represents foods as complete entities rather than decomposing them into molecular components. Each food is modeled as a single graph node connected through typed relational edges to its constituent compounds and other biological entities, enabling simultaneous capture of compound-mediated pharmacological mechanisms and higher-order semantic relationships. This addresses the fundamental misalignment between computational prediction methods and clinical practice, where dietary recommendations are provided based on foods. By integrating FooDB, DRKG, and CTD into a unified knowledge graph (41,698 entities, 71 relation types, and 1.4 million triplets) and employing cross-attention mechanisms with R-GCNs, KG-DFI captures clinically relevant DFI patterns that component-based approaches cannot adequately model.

This predictive capacity does not depend primarily on fine-grained compound-level mechanistic detail. Because each food is linked into the knowledge graph only through its constituent compounds, the model’s predictive signal appears to derive primarily from aggregating a food’s overall compound profile, supporting the entity-level, whole-food framing adopted here.

Although KG-DFI, GAT+CrossAttention, and GAT-only achieved statistically comparable predictive performance, they differ fundamentally in where attention is applied. GAT performs attention during neighborhood aggregation, whereas our cross-attention module models conditional interactions between the final food and drug representations after graph encoding. Because these 2 mechanisms operate at different semantic levels, cross-attention complements rather than duplicates R-GCN. Furthermore, R-GCN naturally preserves relation-specific transformations across heterogeneous biomedical edges while scaling to the complete graph without neighborhood sampling, making it a more suitable backbone for our knowledge graph. R-GCN is additionally trained end-to-end with the classification objective, unlike the knowledge graph embedding baselines’ decoupled pretraining stage, and its layer-wise message passing supports the pathway-level interpretability.

Existing DFI prediction methods have largely operated at the molecular constituent level. DFinder queried pharmacological databases for interactions between individual food compounds and drugs, and FDmine extended this via large-scale mining of compound-level interactions [61,62]. These approaches require decomposing each food into its constituent compounds, introducing computational overhead proportional to compound inventory and potentially missing synergistic effects across compounds. By contrast, KG-DFI represents each food as a single entity within a heterogeneous knowledge graph, capturing both compound-mediated mechanisms and higher-order semantic relationships. Direct numerical comparison with DFinder or FDmine is not feasible given differences in dataset scope and evaluation protocols; however, KG-DFI’s food-level representation aligns with the granularity of clinical dietary guidance, and its relational structure enables multihop inference across pharmacological pathways not accessible to single-source compound queries.

To characterize what pharmacological structure the model’s R-GCN-based graph representations encode, we performed a secondary graph traversal analysis confirming that multihop pathways through shared biological targets, enzymatic nodes, and transporter proteins account for a substantial proportion of high-confidence predictions. For example, grapefruit contains constituent compounds connected to the CYP3A4 enzyme node via 3-hop paths, and CYP3A4 itself is reachable from 756 of the 894 food entities in the knowledge graph (84.6%) through at least 1 such compound-mediated path, demonstrating that the knowledge graph encodes known pharmacological pathways within its heterogeneous relational structure and that KG-DFI can identify nonobvious, cross-database interaction signals that single-source approaches cannot capture.

An examination of misclassified cases in Table 4 reveals that directional prediction errors are not distributed uniformly across drugs: 5 of the 7 directional errors—all instances in which KG-DFI overestimated interaction risk—involve a single drug, ferric maltol, which we found to be markedly more sparsely connected in the knowledge graph than 2 comparably classified iron-based drugs, notably lacking the drug–drug interaction edges both comparators have; this pattern is not attributable to training supervision and does not extend to the remaining 2 directional errors, which involve well-connected drugs and represent underestimation rather than overestimation. We present this as a plausible, case-grounded mechanism rather than a statistically generalized finding and note that competing food side effects (e.g., vitamin C versus polyphenol effects on iron bioavailability) and label noise in the training data likely compound such errors further. A practical implication is that predictions involving drugs with unusually sparse knowledge graph connectivity may warrant lower-confidence flagging in downstream clinical use; we identify connectivity-aware confidence estimation as a direction for future work. We therefore position KG-DFI as a first-line screening tool for candidate interactions warranting further clinical evaluation, rather than a definitive classifier of interaction directionality.

From a clinical perspective, predicting DFIs at the food level directly aligns with how clinicians provide dietary guidance. The KG-DFI framework could be integrated into electronic health record systems or clinical decision support tools to alert prescribers when a patient’s typical diet may interfere with their medication regimen. As knowledge graph databases expand to include more foods, drugs, and pharmacokinetic parameters, the framework’s coverage and clinical utility are expected to improve. Collaborative curation of high-quality DFI data would further strengthen model reliability.

However, our model has several limitations that require consideration. First, while the model accurately identifies whether DFIs occur (binary classification), the model may misclassify interaction types (multiclass prediction). For instance, ferric maltol interactions with vitamin C-rich foods (silver linden, cabbage) were predicted to be positive based on learned vitamin C–iron synergy [49–51], but the literature revealed negative effects due to dominant flavonoid chelation and food matrix interference [52–55]. Nevertheless, this literature confirms interactions between ferric maltol and these foods, indicating that the model successfully detects interactions even when misclassifying the interaction direction. This limitation arises from insufficient or imbalanced training data on competing interaction mechanisms (e.g., flavonoid–iron chelation versus vitamin C–iron enhancement) in the knowledge graph, which prevents the model from learning the relative importance of opposing effects. Second, performance on rare classes remains suboptimal, particularly for “harmful” interactions (2.4% of the dataset). Despite the implementation of focal loss, sensitivity for clinically critical interactions remains low. Notably, when the model failed to identify a Harmful interaction in the held-out test set, it consistently defaulted toward a reassuring label (Possible or Positive) rather than a cautionary one (Negative); a specific safety-relevant limitation that warrants targeted attention in future work. Given that only 6 Harmful and 12 Negative instances were available in the held-out test set, targeted data augmentation for these rare classes could further improve rare-class sensitivity beyond what focal loss alone achieves. A positive-unlabeled learning formulation—which explicitly models the possibility that some pairs currently labeled Possible reflect under-annotated Negative or Harmful interactions—offers a complementary direction. Complementing these approaches, an asymmetric, cost-sensitive loss that penalizes Harmful→Possible/Positive misclassifications more heavily than Harmful→Negative ones would directly address this safety-relevant error pattern. Third, predictive capacity depends on the connectivity of the knowledge graph. Indeed, disconnected entities cannot be effectively evaluated regardless of model sophistication. Meanwhile, continued database integration and enhanced entity-mapping strategies are essential for expanding coverage. Fourth, the framework does not incorporate quantitative factors that influence interaction outcomes. The knowledge graph represents food–compound relationships as binary associations, lacking quantitative information such as compound concentrations, dose-response relationships, or interaction magnitudes. For instance, vitamin C content in cabbage ranges from 12.0 to 112.5 mg/100 g [50], quercetin concentrations vary substantially by variety, and ferric maltol shows 5-fold bioavailability differences based on food matrix presence. These quantitative factors critically determine interaction strength and clinical relevance but are not captured in current binary graph representations. Integration of quantitative data, including compound concentrations, pharmacokinetic parameters, and dose–response profiles, would enable more accurate prediction of the magnitude and clinical impact of interactions. This limitation is compounded by the source database’s own annotation scheme. Because DDID assigns interaction labels based solely on whether an interaction is recorded, without a minimum dose or concentration threshold, a single documented occurrence of an interaction is labeled identically to a well-established, high-magnitude one. As a result, the model cannot currently distinguish clinically trivial from clinically meaningful instances within a given interaction class. A representative example is the grapefruit–CYP3A4 interaction: This well-characterized effect requires substantial grapefruit consumption to become clinically meaningful. Yet, the model may flag structurally similar food–drug pairs as interacting without any indication of the consumption level at which the interaction becomes relevant, underscoring the need for future integration of quantitative pharmacokinetic and food-matrix data. A related limitation concerns the negative sampling strategy: Drug–food pairs absent from DDID are treated as negative examples under a closed-world assumption. This may introduce false-negative contamination if the absence of documentation reflects incomplete literature coverage rather than a true absence of interaction. Systematic evaluation of alternative negative-sampling strategies, such as structurally similar hard negatives, class-stratified negatives, or filtering candidate negatives against external pharmacovigilance sources, is identified as a priority direction for assessing and improving model robustness. Fifth, the current framework operates in a transductive setting, in which entity embeddings are derived from the full knowledge graph prior to train–test partitioning. Consequently, the model cannot generate predictions for entirely new drug or food entities that were absent from the knowledge graph at training time. Relatedly, the present evaluation uses a random pair-level split in which entities appearing in the test set were also observed during training. This is expected to yield more optimistic performance estimates than a food-wise, drug-wise, or fully cold-start split, in which test entities are entirely withheld from training. Cold-start evaluation is identified as an important direction for future work to more rigorously assess generalizability to novel entities.

Several directions remain important for future development of this framework. The current model outputs logit-derived probability estimates that have not undergone formal calibration; a diagnostic analysis confirms that the model is systematically overconfident, especially for the rare Negative and Harmful classes. Methods such as temperature scaling, Platt scaling, or Monte Carlo dropout could be incorporated to produce well-calibrated uncertainty estimates actionable in clinical decision support. Furthermore, the present KG-DFI framework operates in a transductive setting, and performance estimates should be interpreted within this context; future inductive extensions capable of producing embeddings for entities absent from the training graph would substantially expand generalizability. Finally, deployment of a web-accessible query interface remains a priority direction, allowing clinicians and pharmacists to interactively query the model for specific drug–food pairs without requiring local installation.

## Conclusion

This study proposed a KG-DFI prediction model that represents foods as complete entities for DFI prediction based on the food to address the fundamental gap between component-based prediction approaches and clinical practice. The ability of our model to predict interactions without molecular decomposition can help develop practical decision-support systems that align with how healthcare providers deliver dietary guidance to patients. Moreover, by integrating major biomedical databases (FooDB, DRKG, and CTD) into a unified knowledge graph and employing cross-attention mechanisms with R-GCNs, we captured interaction patterns that outperformed existing baseline approaches. Literature validation confirmed the capacity of the model to identify well-characterized interactions and complex multimechanism patterns. These findings enable evidence-based dietary recommendations for individuals on complex medication regimens, contributing to safer therapeutic outcomes and reducing adverse drug events caused by unrecognized DFIs. This work establishes a methodological foundation for predicting DFIs based solely on food.

We anticipate that this work will accelerate further research into DFIs through knowledge graph-based reasoning and ultimately contribute to more reliable dietary recommendations for patients undergoing multifaceted pharmacological treatment.

## Data Availability

The processed datasets and pretrained model weights used in the current research are publicly available at Zenodo (https://doi.org/10.5281/zenodo.21275098). The source code is publicly available at https://github.com/bmil-jnu/KG-DFI.
