# Supplementary material for: KG-DFI: A Prediction of Drug–Food Interactions Based on Knowledge Graph Embedding
Source: Comput Struct Biotechnol J. 2026 Aug 18;35(1):0205. doi: 10.34133/csbj.0205 (PMC13481788; doi:10.34133/csbj.0205)
Supplement: Supplementary file 1 [file csbj.0205.f1.zip › fig S1 and S2 caption.docx]

Figure S1. Distribution of entities and edges in the customized knowledge graph used in the KG-DFI model. (A) Number of nodes across entity categories, and (B) number of edges across relation types in the constructed knowledge graph.

**Figure S2. Architecture of the R**–**GCN module for heterogeneous knowledge graph representation learning.** (A) Heterogeneous knowledge graph encoding: the knowledge graph is represented as a node feature matrix ($N\times D$), where $N$ denotes the number of entities and $D$ denotes the embedding dimension. (B) Relation-specific neighbor aggregation: each node aggregates information from its neighbors using relation-specific weight matrices, following the update rule in Eq. (1). Stacking three R–GCN layers allows information to propagate across up to three-hop neighborhoods. (C) Multi-hop node embedding: the final node embedding encodes both direct connectivity and multi-hop relational patterns, and serves as input to the cross-attention module.
