# Supplementary material for: KG-DFI: A Prediction of Drug–Food Interactions Based on Knowledge Graph Embedding
Source: Comput Struct Biotechnol J. 2026 Aug 18;35(1):0205. doi: 10.34133/csbj.0205 (PMC13481788; doi:10.34133/csbj.0205)
Supplement: Supplementary file 1 [file csbj.0205.f1.zip › Supplementary File 1.docx]

**Supplementary File 1**

**< Database Curation and Identifier Mapping>**

To construct the customized knowledge graph for drug–food interaction prediction, three public databases—FooDB, the Drug Repurposing Knowledge Graph (DRKG), and the Comparative Toxicogenomics Database (CTD)—were integrated. Because each database uses different entity scopes, identifier systems, and relation schemas, systematic curation was performed prior to integration. This section details the curation criteria, the identifier standardization rules, and the rationale for each step that were summarized in the main text.

**FooDB curation**

FooDB catalogs dietary foods together with their constituent compounds. The curation was restricted to entries under the "foods" category, and the "herbs" category was excluded. This choice reflects the scope of the present work, which targets clinically relevant dietary items consumed as whole foods rather than herbal preparations used for medicinal or supplemental purposes.

Component compounds lacking an International Chemical Identifier (InChI) Key were removed during the curation process. The InChI Key was adopted as the minimum requirement for chemical-structure consistency because it provides a hash-based, structure-derived identifier that allows unambiguous matching of compounds across databases [1]. Compounds without an InChI Key cannot be reliably aligned with entries in DRKG or CTD and were therefore excluded to prevent ambiguous cross-database linkage. After filtering, 98,603 unique food–compound associations remained, comprising 894 food items and 11,580 unique component compounds.

**DRKG drug subgraph extraction**

DRKG integrates biomedical information from six databases (DrugBank, Hetionet, GNBR, STRING, IntAct, and DGIdb) together with literature evidence from COVID-19 studies [2]. Entities in DRKG are labeled with type-specific namespace prefixes (e.g., "Compound::", "Gene::", "Disease::"), and compound entities were identified using the "Compound::" namespace pattern. A drug subgraph was then constructed by retrieving all biological entities and relationships directly connected to these compound entities. This extraction yielded 1,264,354 triplets with 4,687 compound entities, covering interactions with genes, diseases, side effects, anatomical therapeutic chemical (ATC), and pharmacological classes.

**CTD curation and identifier mapping**

The CTD provides mechanistic chemical–gene interaction data, with an initial dataset of 1,048,547 records [3]. Only compounds with CAS registry numbers were retained, as CAS numbers serve as the primary indexing key in CTD and provide a stable reference for cross-database mapping.

To integrate CTD with FooDB and DRKG, CAS registry numbers were mapped to standardized database identifiers: PubChem CID, ChEMBL, and DrugBank. For each compound, the availability of these three identifiers was checked, and a single compound entity ID was assigned according to the following priority: (i) DrugBank identifier, if available; (ii) ChEMBL identifier; and (iii) PubChem CID. DrugBank was prioritized because it provides the most comprehensive pharmacological annotation for approved drugs, which aligns with the clinical focus of this work [4]. ChEMBL was used as the secondary option for its broad coverage of bioactive compounds, and PubChem CID was retained as a fallback to maximize mapping coverage [5, 6]. Gene entities were standardized using NCBI Gene IDs to ensure consistency with the gene identifiers used in DRKG [7].

After filtering and deduplication, 422,276 unique compound–gene interaction triplets spanning 21 mechanistic interaction types were obtained for subsequent connectivity-based integration.

1.	Heller, S.R., et al., *InChI, the IUPAC international chemical identifier.* Journal of cheminformatics, 2015. **7**(1): p. 23.

2.	Zhang, R., et al., *Drug repurposing for COVID-19 via knowledge graph completion.* Journal of biomedical informatics, 2021. **115**: p. 103696.

3.	Davis, A.P., et al., *Comparative toxicogenomics database (CTD): update 2023.* Nucleic acids research, 2023. **51**(D1): p. D1257–D1262.

4.	Wishart, D.S., et al., *DrugBank 5.0: a major update to the DrugBank database for 2018.* Nucleic acids research, 2018. **46**(D1): p. D1074–D1082.

5.	Mendez, D., et al., *ChEMBL: towards direct deposition of bioassay data.* Nucleic acids research, 2019. **47**(D1): p. D930–D940.

6.	Kim, S., et al., *PubChem 2023 update.* Nucleic acids research, 2023. **51**(D1): p. D1373–D1380.

7.	Brown, G.R., et al., *Gene: a gene-centered information resource at NCBI.* Nucleic acids research, 2015. **43**(D1): p. D36–D42.
