# Supplementary material for: KG-DFI: A Prediction of Drug–Food Interactions Based on Knowledge Graph Embedding
Source: Comput Struct Biotechnol J. 2026 Aug 18;35(1):0205. doi: 10.34133/csbj.0205 (PMC13481788; doi:10.34133/csbj.0205)
Supplement: Supplementary file 1 [file csbj.0205.f1.zip › Supplementary File 10.docx]

**Supplementary File 10**

*< Confidence Calibration Analysis >*

This section evaluates whether KG-DFI’s predicted class probabilities reflect the true likelihood of correctness, using the held-out multi-class test set (n = 247) and the same trained model underlying Table 3. For each test sample, confidence is defined as the probability assigned to the predicted (arg max) class. Calibration is assessed via the Expected Calibration Error (ECE) and a reliability diagram, following standard practice for multi-class classifiers.

**Overall calibration**

Across the full test set, mean predicted confidence (0.960) exceeds observed accuracy (0.862) by a substantial margin, and the Expected Calibration Error is 0.0995 (10-bin) — indicating that the model is systematically overconfident. As shown in Figure S6, this overconfidence holds across nearly the entire confidence range: even within the highest-confidence bin (0.9–1.0), which contains the large majority of test samples (217 of 247, 87.9%), observed accuracy (0.908) still falls short of the average stated confidence (0.992) in that bin.

**Figure S6.** Reliability diagram (left) and confidence distribution (right) for the multi-class test set. The dashed diagonal in the left panel indicates perfect calibration; bars falling below the diagonal indicate overconfidence. The right panel shows that predictions are heavily concentrated in the highest-confidence bin.

**Per-class calibration**

Table S11 reports confidence, accuracy, and ECE separately for each of the four interaction classes. Overconfidence is comparatively modest for the majority Possible class but increases substantially for the rarer Negative and Harmful classes, for which the model continues to report high confidence (0.898 and 0.918, respectively) despite accuracy no better than chance among four classes (0.500 for both). Given the very small Negative (n = 12) and Harmful (n = 6) test samples, the specific ECE values for these two classes should be interpreted as an indicative pattern rather than precise estimates; nonetheless, the direction of the trend — confidence remaining high while accuracy degrades for the rarest, most clinically consequential classes — is consistent with the safety-relevant Harmful-class misclassification pattern reported in the main text and in Supplementary File 4.

**Table S11**. Per-class confidence, accuracy, and Expected Calibration Error (5-bin) on the held-out multi-class test set.

| **Class** | **n** | **Mean confidence** | **Accuracy** | **ECE (5-bin)** |
| --- | --- | --- | --- | --- |
| Possible | 182 | 0.966 | 0.912 | 0.054 |
| Positive | 47 | 0.955 | 0.809 | 0.167 |
| Negative | 12 | 0.898 | 0.500 | 0.398 |
| Harmful | 6 | 0.918 | 0.500 | 0.419 |

**Relationship to model deployment**

These findings indicate that the model’s raw softmax outputs should not be interpreted as well-calibrated probabilities, particularly for the Negative and Harmful classes, and motivate incorporating a formal calibration step (e.g., temperature scaling, Platt scaling, or Monte Carlo dropout) prior to any use of confidence scores in clinical decision support, as discussed in the main text.
