# Supplementary material for: KG-DFI: A Prediction of Drug–Food Interactions Based on Knowledge Graph Embedding
Source: Comput Struct Biotechnol J. 2026 Aug 18;35(1):0205. doi: 10.34133/csbj.0205 (PMC13481788; doi:10.34133/csbj.0205)
Supplement: Supplementary file 1 [file csbj.0205.f1.zip › Supplementary File 2.docx]

**Supplementary File 2**

**< DrugBank External Validation: Filtering Criteria and Intermediate Counts>**

This section details the step-by-step filtering procedure applied to DrugBank (version 5.1.5) to construct the external validation reference dataset, including the rationale for each filtering step and the intermediate entry counts that were summarized in the main text.

**DrugBank as the validation resource**

DrugBank is a curated, publicly accessible database that integrates comprehensive information on the chemical, pharmacological, and pharmaceutical properties of drugs, along with detailed annotations of the associated molecular targets. DrugBank categorizes drugs by regulatory status and development stage, including approved, investigational, nutraceutical, experimental, and other categories. This classification scheme allowed systematic selection of compounds with verified clinical status, making DrugBank suitable as an external reference for evaluating the generalizability and clinical relevance of the predicted drug–food interactions.

**Four-step filtering procedure**

Starting from 17,430 total entries in DrugBank, a four-step filtering procedure was applied to extract oral drugs approved by the United States Food and Drug Administration (FDA) and to remove non-specific food category labels from the resulting food–drug pairing pool.

**Step 1. Approval status, marketing availability, and route of administration.** Entries were first filtered to retain drugs that were (i) approved, (ii) marketed in the United States, and (iii) administered via the oral route. This step yielded 1,162 drugs.

**Step 2. Cross-referencing with the DrugBank structure links file.** The remaining entries were cross-referenced with the DrugBank "structure links" file, which provides chemical structure identifiers and cross-database links (e.g., DrugBank ID, CAS Number, InChIKey, PubChem CID, ChEMBL ID) together with a "Drug Groups" annotation for each compound. Only entries whose "Drug Groups" values corresponded to approved-related classifications were retained. The accepted classifications were: "approved", "approved; investigational", "approved; vet_approved", and "approved; nutraceutical". This step yielded 986 unique FDA-approved oral drugs, corresponding to approximately 5.8% of all DrugBank compounds.

**Step 3. Mapping to the customized knowledge graph.** The 986 drugs were mapped to the customized knowledge graph, which contains 41,698 nodes. Of these, 925 drugs were matched with DrugBank IDs present in the compound entities of the knowledge graph, while the remaining 61 drugs were excluded because their DrugBank IDs were not registered in the compound namespace of the underlying DRKG source. The knowledge graph contains 894 food entities after the herbs exclusion described in Supplementary File 1; an additional food-side filtering step, described below, was applied before pairing with the 925 mapped drugs.

**Step 4. Food-side category-level filtering.** Manual inspection of the 894 food entities identified five entries corresponding to broad, non-specific category labels rather than individually consumed foods: “beverage” (FOOD00870), “fruits” (FOOD00871), “Mollusks” (FOOD00868), “Herbs and Spices” (FOOD00866), and the “Grapefruit/Pummelo hybrid” grouping (FOOD00255, distinct from the specific “Grapefruit” entity, FOOD00256, which was retained). These five entries were excluded using the same rationale applied to the herbs category in Supplementary File 1—namely, that they denote taxonomic or commercial groupings rather than foods as clinically consumed—reducing the food-entity pool from 894 to 889. The “Alcoholic beverages” entry (FOOD00854) was deliberately retained despite superficially resembling a category label, because it denotes a specific, clinically well-defined dietary pattern with distinct pharmacological relevance (e.g., alcohol–sedative and alcohol–antibiotic interactions) rather than a generic placeholder; this entity underlies the alcoholic beverages case studies discussed in Table 4 and Supplementary File 8. Applying this filtering step yielded the final 925 × 889 = 822,325 drug–food pairs used for external validation.

**Rationale for restricting to FDA-approved oral drugs**

Restricting the validation to FDA-approved oral drugs ensured that the analysis reflected clinically relevant pharmacotherapies with well-established safety and pharmacokinetic profiles. This strategy enhances the interpretability of predicted drug–food interactions and minimizes biases arising from compounds with limited clinical evidence.
