# Supplementary material for: KG-DFI: A Prediction of Drug–Food Interactions Based on Knowledge Graph Embedding
Source: Comput Struct Biotechnol J. 2026 Aug 18;35(1):0205. doi: 10.34133/csbj.0205 (PMC13481788; doi:10.34133/csbj.0205)
Supplement: Supplementary file 1 [file csbj.0205.f1.zip › Supplementary File 3.docx]

**Supplementary File 3**

**< Model Architecture, Training Configuration, and Evaluation Metrics>**

This section provides the detailed model architecture, training configuration, and formal definitions of the evaluation metrics used to assess the KG-DFI model, which were summarized in the main text.

**Network architecture**

The concatenated attention-enhanced food and drug embeddings were processed through a multi-layer perceptron (MLP) consisting of four fully-connected layers with dimensions 512 → 128 → 128 → 1. Rectified linear unit (ReLU) activations and batch normalization were applied to intermediate layers, and dropout regularization with dropout probability p = 0.3 was applied between MLP layers to mitigate overfitting. The final sigmoid layer produced interaction probabilities for binary classification. Training was performed with the Adam optimizer (learning rate = 5e-4), batch size 64, early stopping with patience 15 epochs, and a learning rate scheduler.

**Focal Loss parameters**

The Focal Loss formulation (given in the main text) was applied with α = 0.25 and γ = 1.0, as determined by the grid search reported in Supplementary File 5. The weighting factor α = 0.25 reduces the contribution of easily classified majority-class samples, and the focusing parameter γ = 1.0 down-weights well-classified examples so that learning concentrates on harder, minority-class cases.

**Formal definitions of evaluation metrics**

For binary classification, let TP, TN, FP, and FN denote the numbers of true positives, true negatives, false positives, and false negatives, respectively.

Accuracy measures the overall proportion of correct predictions:

$$Accuracy=\frac{TP+TN}{TP+TN+FP+FN}$$

Precision quantifies the proportion of true positive predictions among all positive predictions:

$$Precision=\frac{TP}{TP+FP}$$

Recall (sensitivity) measures the proportion of actual positive cases correctly identified:

$$Recall =\frac{TP}{TP + FN}$$

F1-score provides the harmonic mean of precision and recall, balancing both metrics:

$$F1-Score=2\times\frac{Precision\times Recall}{Precision+Recall}$$

Weighted metrics are used to address class imbalance in multi-class evaluation. Each per-class metric is weighted by the number of true instances (support) of that class and averaged across classes:

$$\text{Metric}_{\text{weighted}}=\frac{\sum_{i=1}^{k} \text{support}_{i}\times\text{Metric}_{i}}{\sum_{i=1}^{k} \text{support}_{i}}$$

where $k$ denotes the number of classes and $\text{support}_{i}$ denotes the number of true instances in class $i$.

Cohen’s kappa ($\kappa$) measures classification agreement corrected for chance:

$$\kappa=\frac{p_{o}-p_{e}}{1-p_{e}}$$

where $p_{o}$ is observed proportion of agreement and $p_{e}$ is the proportion of agreement expected by chance, computed from the marginal distributions of the predictions and the true labels. A value of $\kappa$ = 1 indicates perfect agreement, $\kappa$ = 0 indicates agreement no better than chance, and negative values indicate disagreement worse than chance.
