# Supplementary material for: KG-DFI: A Prediction of Drug–Food Interactions Based on Knowledge Graph Embedding
Source: Comput Struct Biotechnol J. 2026 Aug 18;35(1):0205. doi: 10.34133/csbj.0205 (PMC13481788; doi:10.34133/csbj.0205)
Supplement: Supplementary file 1 [file csbj.0205.f1.zip › Supplementary File 4.docx]

**Supplementary File 4**

**< Confusion Matrix Analysis of the KG-DFI Classifier >**

This section presents the full confusion matrix of the KG-DFI model evaluated on the held-out test set under the best-performing hyperparameter configuration. The matrix supplements the per-class performance metrics reported in the main text and provides a detailed view of prediction patterns across the four drug–food interaction (DFI) categories.

**Confusion matrix**

**Figure S3.** Confusion matrix of the KG-DFI model on the held-out test set. (Left) Absolute prediction counts for each true label and predicted label pair. (Right) Row-normalized confusion matrix representing per-class recall. Rows correspond to true labels and columns to predicted labels. The four classes are Possible, Positive, Negative, and Harmful.

**Per-class prediction analysis**

**Table S1. Per-class precision, recall, and F1-score on the held-out test set.**

| **Class** | **n (test)** | **Precision** | **Recall** | **F1-score** |
| --- | --- | --- | --- | --- |
| Possible | 182 | 0.9326 | 0.9121 | 0.9222 |
| Positive | 47 | 0.7755 | 0.8085 | 0.7917 |
| Negative | 12 | 0.6000 | 0.5000 | 0.5455 |
| Harmful | 6 | 0.3000 | 0.5000 | 0.3750 |

Possible class (recall = 0.91, n = 182 test samples). The Possible class achieved the highest recall among all four categories. Of 182 test samples, 166 were correctly classified, with minor misclassifications distributed across Positive (7), Negative (4), and Harmful (5).

Positive class (recall = 0.81, n = 47 test samples). The Positive class achieved a recall of 0.81. Eight samples (17%) were misclassified as Possible, reflecting the semantic overlap between Possible and Positive DFI categories, both of which describe non-harmful food–drug associations. One sample was misclassified as Harmful.

Negative class (recall = 0.50, n = 12 test samples). The Negative class showed the lowest recall, with 6 of 12 test samples correctly classified. Remaining samples were attributed to Possible (3), Positive (2), or Harmful (1). The small number of Negative test samples (n = 12) limits the reliability of the per-class estimate for this category.

Harmful class (recall = 0.50, n = 6 test samples). The Harmful class achieved a recall of 0.50, with 3 of 6 samples correctly identified. The remaining samples were misclassified as Possible (1) or Positive (2). Given the clinical significance of Harmful interactions, Focal loss with concentration parameter γ = 1.0 was applied during training to increase the relative attention paid to minority-class examples. Harmful class recall was specifically monitored as a secondary evaluation criterion alongside the primary weighted F1 metric.

**Interpretation of class imbalance effects**

The variation in per-class recall across DFI categories reflects the inherent class imbalance in curated interaction datasets. The Possible class, which constitutes the largest proportion of training and test samples, achieves the highest recall (0.91), while the minority Harmful and Negative classes show lower performance (recall = 0.50 for both). This pattern is consistent with established challenges in multi-class imbalanced classification and motivated the adoption of weighted F1 as the primary evaluation metric, which accounts for class frequency differences during model selection and cross-method comparison.

**Adjacent versus erratic misclassification patterns**

To assess whether misclassifications tend to occur between semantically similar categories or across unrelated categories, we grouped the four DFI classes into two tiers based on their clinical framing: a non-harmful tier (Possible, Positive) and a clinically concerning tier (Negative, Harmful). Under this grouping, each of the 34 misclassified test samples was categorized as an adjacent error (misclassification within the same tier: Possible↔Positive or Negative↔Harmful) or an erratic error (misclassification across tiers). Adjacent errors accounted for 16 of 34 misclassifications (47.1%: Possible→Positive = 7, Positive→Possible = 8, Negative→Harmful = 1), and erratic errors accounted for the remaining 18 of 34 (52.9%: Possible→Negative = 4, Possible→Harmful = 5, Positive→Harmful = 1, Negative→Possible = 3, Negative→Positive = 2, Harmful→Possible = 1, Harmful→Positive = 2). Given the small number of test samples in the Negative (n = 12) and Harmful (n = 6) classes, these proportions should be interpreted with caution, as reassigning a single sample shifts each percentage by several points.

The most clinically relevant erratic pattern involves the Harmful class specifically. Of the 3 missed Harmful test samples, none were misclassified as Negative (which would still signal a clinically relevant concern), and all 3 were instead misclassified as Possible (1) or Positive (2) — categories that convey no risk or a beneficial interaction. This indicates that when the model fails to identify a Harmful interaction, it tends to default toward a reassuring rather than cautionary label. We identify this as a specific safety-relevant limitation of the current model, distinct from the general Harmful-class sensitivity limitation discussed in the main text, and one that warrants targeted attention (e.g., asymmetric misclassification costs during training) in future work.

**Cross-attention modification magnitude by interaction class**

We additionally computed the cross-attention module’s per-class modification magnitude, using the same model and held-out test set underlying Table 3: the attention-output norm (the magnitude of drug-context information injected into the food representation) and the total food-embedding modification (Table S2). The attention-output norm is somewhat lower for the Negative class than for the other three classes; the food-embedding modification measure shows no clear class-dependent trend. Because cross-attention operates on food and drug representations already aggregated by R-GCN into single entity-level vectors, the attention weight itself cannot be attributed to specific compounds or genes within the graph. The clearest evidence that cross-attention contributes beyond added model capacity remains its removal causing Cohen’s kappa to fall from 0.6646 to 0.1166 (Table 3).

**Table S2. Cross-attention modification magnitude by interaction class.**

| **Class** | **n (test)** | **Attention-output norm (mean ± SD)** | **Food-embedding modification (mean ± SD)** |
| --- | --- | --- | --- |
| Possible | 182 | 12.35 ± 1.91 | 14.74 ± 3.50 |
| Positive | 47 | 12.34 ± 4.37 | 12.88 ± 3.16 |
| Negative | 12 | 11.18 ± 1.56 | 13.58 ± 4.29 |
| Harmful | 6 | 12.21 ± 2.00 | 12.27 ± 1.54 |
