# Supplementary material for: KG-DFI: A Prediction of Drug–Food Interactions Based on Knowledge Graph Embedding
Source: Comput Struct Biotechnol J. 2026 Aug 18;35(1):0205. doi: 10.34133/csbj.0205 (PMC13481788; doi:10.34133/csbj.0205)
Supplement: Supplementary file 1 [file csbj.0205.f1.zip › Supplementary File 5.docx]

**Supplementary File 5**

**< Hyperparameter Sensitivity Analysis of the KG-DFI Model >**

This section presents a systematic hyperparameter sensitivity analysis of the KG-DFI model, examining the effects of embedding dimension, number of R-GCN layers, focal loss concentration parameter (γ), and number of cross-attention heads on validation-set weighted F1. The analysis was conducted to select and justify the final model configuration reported in the main text.

**Grid search protocol**

A grid search was conducted over the following hyperparameter ranges: embedding dimension ∈ {64, 128, 256}, number of R-GCN layers ∈ {1, 2, 3, 4}, focal loss γ ∈ {0.5, 1.0, 2.0, 3.0}, and number of cross-attention heads ∈ {2, 4, 8}. For each configuration, the model was trained under identical conditions using the same random seed, training schedule, and data split, and was evaluated on the validation set using weighted F1. The configuration that maximized validation-set weighted F1 was subsequently evaluated on the held-out test set.

**Hyperparameter sensitivity results**

**Figure S4.** Hyperparameter sensitivity analysis for KG-DFI. (A) Heatmap of validation-set weighted F1 as a function of embedding dimension and number of R-GCN layers. Each cell reports the best weighted F1 across all values of γ and attention heads. The selected configuration is marked with a star. (B) Weighted F1 as a function of focal loss γ with embedding dimension = 256, R-GCN layers = 3, and attention heads = 4 held fixed. (C) Weighted F1 as a function of the number of cross-attention heads with embedding dimension = 256, R-GCN layers = 3, and γ = 1.0 fixed. Red stars indicate the selected value for each hyperparameter.

**Embedding dimension and number of R-GCN layers (Figure S4. (A))**

Panel A presents a heatmap of validation-set weighted F1 as a function of embedding dimension and number of R-GCN layers, with the best performance over γ and attention heads reported for each cell. The highest performance (weighted F1 = 0.882) was achieved with an embedding dimension of 256 and 3 R-GCN layers. Performance across the full grid remained relatively stable (range: 0.863–0.882), but consistently favored larger embedding dimensions and deeper R-GCN encoders, indicating that additional representational capacity and multi-hop neighborhood aggregation provide a modest benefit for capturing structural information from the heterogeneous knowledge graph in this task.

**Focal loss γ (Figure S4. (B))**

Panel B shows the effect of the focal loss concentration parameter γ on validation-set weighted F1, with embedding dimension = 256, R-GCN layers = 3, and attention heads = 4 fixed. A γ value of 1.0 yielded the highest performance (weighted F1 = 0.882). Performance was substantially lower at γ = 0.5 (weighted F1 = 0.764) and declined moderately for γ ≥ 2.0 (weighted F1 = 0.838 at γ = 2.0; 0.830 at γ = 3.0). This pattern indicates that a moderate degree of down-weighting of easy examples (γ = 1.0) is beneficial for addressing class imbalance in the DFI prediction task: insufficient down-weighting (γ = 0.5) allows the abundant easy majority-class examples to dominate the loss, whereas excessive concentration on hard examples (γ ≥ 2.0) degrades overall predictive performance.

**Number of cross-attention heads (Figure S4. (C))**

Panel C shows the effect of the number of cross-attention heads on validation-set weighted F1, with embedding dimension = 256, R-GCN layers = 3, and γ = 1.0 fixed. The optimal configuration used 4 attention heads (weighted F1 = 0.882). Reducing to 2 heads (weighted F1 = 0.868) or increasing to 8 heads (weighted F1 = 0.855) resulted in performance reductions. These differences suggest that the cross-attention module benefits from a moderate number of attention subspaces, with too few or too many heads modestly degrading performance within the tested range.

**Selected configuration**

Based on the grid search results, the final KG-DFI model was trained with the following configuration: embedding dimension = 256, number of R-GCN layers = 3, focal loss γ = 1.0, and number of cross-attention heads = 4. This configuration achieved a validation-set weighted F1 of 0.882 during the grid search. The model was subsequently retrained under this configuration for final evaluation, achieving weighted F1 = 0.8801, AUROC = 0.9163, AUPRC = 0.9234, and Cohen’s κ = 0.6646 on the held-out test set, as reported in the main text (Table 3).

**Traditional machine learning baseline hyperparameters**

Hyperparameters for the traditional machine learning baselines (SVM, Random Forest, XGBoost, and LightGBM) were selected via five-fold cross-validation grid search on the training set, using the same KG-derived entity embeddings as input features. The optimal configuration identified for each baseline is summarized in Table S3. These configurations are reported to confirm that each baseline was reasonably tuned prior to comparison with KG-DFI in Table 2.

**Table S3. Optimal hyperparameters for traditional machine learning baselines, selected by five-fold cross-validation grid search.**

| **Model** | **Optimal hyperparameters** |
| --- | --- |
| SVM | Kernel: RBF; C = 1.0; γ = ‘scale’ |
| Random Forest | 100 estimators; max_depth = None; min_samples_split = 2 |
| XGBoost | learning_rate = 0.1; n_estimators = 100; max_depth = 6 |
| LightGBM | num_leaves = 31; learning_rate = 0.05; n_estimators = 100 |
