# Supplementary material for: KG-DFI: A Prediction of Drug–Food Interactions Based on Knowledge Graph Embedding
Source: Comput Struct Biotechnol J. 2026 Aug 18;35(1):0205. doi: 10.34133/csbj.0205 (PMC13481788; doi:10.34133/csbj.0205)
Supplement: Supplementary file 1 [file csbj.0205.f1.zip › Supplementary File 6.docx]

**Supplementary File 6**

**< Knowledge Graph Component Ablation Study >**

This section presents a knowledge graph (KG) component ablation study evaluating the contribution of the CTD (Comparative Toxicogenomics Database) relation layer to overall DFI prediction performance. To isolate the effect of CTD, two model configurations were trained and evaluated: (1) KG-DFI trained on the full KG (FooDB + DRKG + CTD), and (2) KG-DFI trained on a reduced KG from which all CTD-derived edges were removed (FooDB + DRKG only). Both configurations used an identical model architecture, training procedure, and held-out test set (n = 171).

**Ablation setup**

The full KG (FooDB + DRKG + CTD) contains 71 unique relation types (142 bidirectional) covering 2,895,934 edges across 41,698 entities. Removing CTD yields a reduced KG with 50 unique relation types (100 bidirectional) and 1,362,957 unique edges (2,725,914 bidirectional). Both configurations shared the same entity set, training split (train: 1,310 / val: 158 / test: 171), model architecture, hyperparameters (embedding dimension = 256, R-GCN layers = 3, focal loss γ = 1.0, cross-attention heads = 4), optimizer, early stopping rule (patience = 20), and random seed (seed = 42).

**Ablation results**

**Table S4.** KG component ablation: performance comparison of KG-DFI with and without CTD relation edges..

| **Configuration** | **Relations** | **F1** | **AUROC** | **AUPRC** | **Kappa** |
| --- | --- | --- | --- | --- | --- |
| FooDB + DRKG (No CTD) | 50 | 0.8734 | 0.9263 | 0.9249 | 0.6453 |
| **FooDB + DRKG + CTD (full)** | **71** | **0.8801** | **0.9163** | **0.9234** | **0.6646** |
| Δ (Full − No CTD) | — | +0.0067 | −0.0100 | −0.0015 | +0.0193 |

**Bootstrap confidence interval for AUROC difference**

To assess whether the AUROC change attributed to CTD (ΔAUROC = −0.0100) represents a statistically meaningful difference, a non-parametric bootstrap procedure was applied (N = 10,000 iterations, n = 171 test samples). The 95% bootstrap confidence interval for the AUROC difference (Full − No CTD) was [−0.0283, +0.0068]. Because this interval includes zero, the AUROC difference is not statistically significant at the 0.05 level. Similarly, the F1 improvement (ΔF1 = +0.0067) and Kappa improvement (ΔKappa = +0.0193) are small in magnitude.

**Interpretation**

The ablation results indicate that CTD provides a modest and statistically non-significant change in AUROC (−0.0100, 95% CI [−0.0283, +0.0068]) while yielding small improvements in F1 (+0.0067) and Kappa (+0.0193). This pattern is consistent with CTD providing complementary biological signal—particularly for mechanistic drug–gene–food pathways—that improves overall classification consistency without substantially altering ranking-based metrics. CTD’s primary contribution is to enrich the KG with directed compound–gene interaction annotations (21 unique CTD relation types), supporting the multi-hop reasoning paths described in Supplementary File 8.

**Food–compound connectivity and the role of CTD**

Food entities in the knowledge graph connect to the rest of the graph exclusively through FooDB’s food–compound (CONTAINS) relation: of 1,447,967 total triples, all 98,603 triples incident on a Food:: entity (894 unique food entities) are CONTAINS relations, and no other relation type touches a food node directly. Predictive signal for food entities is therefore necessarily mediated through the compounds a food contains.

Among the 12,125 unique compounds contributed by food entities via CONTAINS, 18,365 of their outgoing edges reach Gene:: entities. Of these food–compound–gene edges, 15,875 (86.4%) are CTD relations and 2,490 (13.6%) are GNBR literature-mined relations contributed by DRKG. Removing CTD from the knowledge graph (the “No CTD” configuration in Table S4) therefore removes the large majority of this compound–gene mechanistic detail while leaving the CONTAINS-based aggregation structure—and a smaller residual set of GNBR-derived gene links—unchanged. That the resulting performance change was modest and statistically non-significant (see Bootstrap confidence interval above) indicates that the model’s predictive signal for food–drug interactions derives primarily from aggregating a food’s full compound profile into a single entity-level representation, rather than from the fine-grained compound–gene mechanistic detail that CTD predominantly supplies.
