# Supplementary material for: KG-DFI: A Prediction of Drug–Food Interactions Based on Knowledge Graph Embedding
Source: Comput Struct Biotechnol J. 2026 Aug 18;35(1):0205. doi: 10.34133/csbj.0205 (PMC13481788; doi:10.34133/csbj.0205)
Supplement: Supplementary file 1 [file csbj.0205.f1.zip › Supplementary File 7.docx]

**Supplementary File 7**

**< Comprehensive Baseline Comparison >**

This section provides a comprehensive comparison of KG-DFI against six baseline models: Graph Attention Network (GAT), and four knowledge graph embedding (KGE) methods—TransE, DistMult, ComplEx, and RotatE—together with CompGCN. All models were evaluated on the same held-out test set (n = 171) under the same data split (70% / 10% / 10% train/val/test fixed across all runs). To ensure a statistically reliable comparison, each model was trained and evaluated over five independent random seeds (seeds: 42, 123, 456, 789, 999), and performance is reported as mean ± standard deviation across seeds.

**Baseline model descriptions**

KG-DFI (R-GCN, this work) uses a relational graph convolutional network (R-GCN) encoder on the full heterogeneous KG followed by a cross-attention module and MLP classifier. It is trained end-to-end on the DFI prediction objective without a separate pre-training phase.

GAT baseline replaces the R-GCN encoder with a Graph Attention Network (GATConv) while keeping the same cross-attention module and MLP classifier. Because full-batch GAT on the 2.9M-edge KG exceeds GPU memory limits (~39 GB), neighborhood sampling (fanout = 50 neighbors per node) was applied. This memory constraint is itself indicative of a scalability advantage of R-GCN for large heterogeneous graphs.

GAT (no cross-attn) removes the cross-attention module from the GAT baseline above, connecting the GAT encoder output directly to the MLP classifier, to test whether GAT’s native attention mechanism makes a separate cross-attention stage redundant (see “KG-DFI vs. GAT-only” below).

KGE baselines (TransE, DistMult, ComplEx, RotatE) follow a pre-train then fine-tune paradigm: entity embeddings are first pre-trained on the full KG using the respective scoring function (100 epochs, batch size 4,096), then the frozen entity embeddings of food and drug entities are used as inputs to the same MLP classifier used in KG-DFI. ComplEx entity representations use real-valued embeddings of dimension 256 derived from the complex-valued optimization.

CompGCN (Vashishth et al., 2020) is a composition-based GNN for heterogeneous KGs that jointly learns entity and relation representations. CompGCN is trained end-to-end on the DFI prediction task using the same MLP classifier.

**Results**

**Table S5.** Comprehensive baseline comparison.

| **Model** | **Paradigm** | **Params** | **F1** | **AUROC** | **AUPRC** | **Kappa** |
| --- | --- | --- | --- | --- | --- | --- |
| **KG-DFI (R-GCN)** | **End-to-end** | **26.77M** | **0.8768±0.0049** | **0.9314±0.0077** | **0.9325±0.0045** | **0.6538±0.0116** |
| GAT | End-to-end | 13.02M | 0.8738±0.0139 | 0.9431±0.0109 | 0.9407±0.0077 | 0.6557±0.0326 |
| GAT (no cross-attn) | End-to-end | 12.82M | 0.8844±0.0115 | 0.9338±0.0099 | 0.9341±0.0039 | 0.6762±0.0331 |
| ComplEx-DFI | Pre-train + FT | 10.84M† | 0.8752±0.0089 | 0.9300±0.0087 | 0.9259±0.0072 | 0.6511±0.0249 |
| RotatE-DFI | Pre-train + FT | 10.84M† | 0.8551±0.0175 | 0.9278±0.0066 | 0.9323±0.0032 | 0.6189±0.0378 |
| DistMult-DFI | Pre-train + FT | 10.84M† | 0.8470±0.0167 | 0.9214±0.0080 | 0.9160±0.0066 | 0.5970±0.0412 |
| TransE-DFI | Pre-train + FT | 10.84M† | 0.8037±0.0176 | 0.8733±0.0086 | 0.8973±0.0062 | 0.5001±0.0311 |
| CompGCN-DFI | End-to-end | 12.83M | 0.7890±0.0203 | 0.8788±0.0107 | 0.9067±0.0095 | 0.4679±0.0441 |

All metrics are mean ± standard deviation across 5 seeds. Bold = KG-DFI (proposed method). Params = total parameter count. F1 = weighted F1; AUROC = weighted one-vs-rest AUROC; AUPRC = weighted AUPRC; Kappa = Cohen's κ FT = fine-tune.

† KGE model parameter count includes the pre-trained entity embedding matrix (41,698 × 256 = 10.67M) plus a fine-tuning MLP (0.16M);

**Parameter count and model complexity**

KG-DFI (R-GCN encoder, dim=256, L=3) contains 26,773,636 total parameters, including the entity embedding layer (10,674,688; 39.9%), R-GCN encoder (14,551,296; 54.3%), cross-attention module (197,888; 0.7%), and MLP predictor (1,349,764; 5.0%). The cross-attention module contributes only 197,888 parameters (0.7% of total), representing a negligible overhead over the baseline R-GCN + MLP architecture (26,575,748 parameters). GAT contains 13,017,220 total parameters, of which the entity embedding layer accounts for 10,674,688 (82.0%), the GAT encoder for 794,880 (6.1%), the cross-attention module for 197,888 (1.5%, identical in size to KG-DFI’s), and the MLP predictor for 1,349,764 (10.4%). CompGCN-DFI contains 12,832,388 total parameters. KGE baselines (TransE, DistMult, ComplEx, RotatE) each contain approximately 10.84M parameters total, of which 10.67M (98.5%) is the pre-trained entity embedding matrix (dim=256) that is frozen during fine-tuning. The trainable portion during fine-tuning is only the MLP (164,740 parameters). The component-level breakdown for KG-DFI and GAT is summarized in Table S6.

**Table S6. Component-level parameter breakdown for KG-DFI (R-GCN) and the GAT baseline.**

| **Component** | **KG-DFI (R-GCN)** | **GAT baseline** |
| --- | --- | --- |
| Entity embedding | 10,674,688 (39.9%) | 10,674,688 (82.0%) |
| Graph encoder (R-GCN / GAT, 3 layers) | 14,551,296 (54.3%) | 794,880 (6.1%) |
| Cross-attention module | 197,888 (0.7%) | 197,888 (1.5%) |
| MLP predictor | 1,349,764 (5.0%) | 1,349,764 (10.4%) |
| **Total trainable parameters** | **26,773,636 (26.77M)** | **13,017,220 (13.02M)** |

**KG-DFI vs. GAT (end-to-end encoder comparison)**

KG-DFI and GAT were compared under identical conditions: same training split, same cross-attention and MLP modules, same hyperparameters, and 5 independent seeds, trained together in a single execution. (An earlier round of separately-timed comparisons produced inconsistent GAT results; see the reproducibility note below.) Across the four evaluation metrics, GAT scored nominally higher than KG-DFI on AUROC (0.9431±0.0109 vs. 0.9314±0.0077; Welch’s t=−1.96, p=0.09), AUPRC (0.9407±0.0077 vs. 0.9325±0.0045; t=−2.06, p=0.08), and Cohen’s Kappa (0.6557±0.0326 vs. 0.6538±0.0116; t=−0.12, p=0.91), while KG-DFI scored nominally higher only on weighted F1 (0.8768±0.0049 vs. 0.8738±0.0139; t=0.46, p=0.67). None of these four differences reach the conventional 0.05 significance threshold, though the AUROC and AUPRC comparisons approach it; given the limited power of a 5-seed comparison, we do not interpret them as evidence that either architecture achieves superior raw predictive performance, and we report all four metrics rather than emphasizing only the one favorable to KG-DFI. A key practical distinction independent of predictive performance is scalability: R-GCN operates on the full 2.9M-edge graph in batch mode, whereas GAT required neighborhood sampling (fanout = 50) to fit within GPU memory, which may limit its ability to utilize long-range KG structure. R-GCN’s relation-specific weight matrices also distinguish all 73 relation types individually, whereas GAT’s attention mechanism treats all edges uniformly regardless of relation type.

**KG-DFI vs. GAT-only (no cross-attention)**

To test whether GAT’s native attention renders the downstream cross-attention module redundant, we additionally trained a GAT-only variant that removes cross-attention entirely (GAT encoder feeding the MLP predictor directly), under the same conditions and in the same execution as the KG-DFI and GAT+cross-attention runs above. GAT-only achieved F1 = 0.8844±0.0115, AUROC = 0.9338±0.0099, AUPRC = 0.9341±0.0039, and Cohen’s Kappa = 0.6762±0.0331. Relative to GAT+cross-attention, GAT-only scored nominally higher on F1 (t=1.32, p=0.23) and Kappa (t=0.99, p=0.35), and nominally lower on AUROC (t=−1.42, p=0.19) and AUPRC (t=−1.71, p=0.14); relative to KG-DFI, GAT-only scored nominally higher on all four metrics (F1: t=1.38, p=0.22; AUROC: t=0.43, p=0.68; AUPRC: t=0.60, p=0.57; Kappa: t=1.43, p=0.21). None of these eight comparisons reach statistical significance. The inconsistent direction of the GAT-only vs. GAT+cross-attention comparison — cross-attention associated with higher AUROC/AUPRC but lower F1/Kappa — indicates that cross-attention’s marginal effect on a GAT-based encoder is inconclusive at this sample size. This contrasts with R-GCN, for which an existing ablation (Table 3, main text) shows that removing cross-attention causes severe degradation (Cohen’s Kappa falling from 0.6646 to 0.1166): R-GCN’s relation-specific weight matrices provide no pair-level interaction mechanism on their own, so cross-attention is functionally necessary for R-GCN, whereas GAT’s native attention mechanism already performs a related role, making an additional cross-attention stage comparatively redundant for GAT.

**Reproducibility note: GAT result instability across executions**

We observed that GAT-based results vary meaningfully across separately-timed training executions using nominally identical code, hyperparameters, and random seeds. An earlier execution of the GAT+cross-attention comparison reported weighted F1 = 0.8682±0.0189, whereas the execution reported above (run jointly with KG-DFI and GAT-only for internal consistency) gave weighted F1 = 0.8738±0.0139; a separately-timed execution testing only the GAT and GAT-only configurations gave weighted F1 values in the 0.88–0.89 range. This run-to-run variability is consistent with the instability already documented across sampling fanout values below (weighted F1 ranging from 0.8682 to 0.8935 across fanout = 25–150 at a single seed): GAT’s neighborhood-sampling step (fanout = 50, required to fit the model within GPU memory) introduces an additional stochastic element beyond weight initialization, and exact reproducibility of the sampled subgraph across separate executions is not guaranteed even with a fixed random seed. R-GCN’s full-graph, sampling-free processing does not have this source of variability. We note this as an additional, reproducibility-related practical advantage of the R-GCN encoder alongside the scalability, end-to-end training, and interpretability arguments given above, and we report all GAT-related comparisons above from the single execution in which KG-DFI, GAT+cross-attention, and GAT-only were trained together, to ensure internal consistency of the specific comparisons being drawn.

**KG-DFI vs. KGE baselines (pre-train + fine-tune)**

The KGE baselines (TransE, DistMult, ComplEx, RotatE) were tested under a pre-train then fine-tune paradigm, in a separate experiment from the KG-DFI/GAT comparison above. Among KGE methods, ComplEx-DFI performed comparably to KG-DFI across all four metrics: F1 (0.8752±0.0089 vs. 0.8768±0.0049; Welch’s t=0.35, p=0.74), AUROC (0.9300±0.0087 vs. 0.9314±0.0077; t=0.27, p=0.80), AUPRC (0.9259±0.0072 vs. 0.9325±0.0045; t=1.74, p=0.13), and Cohen’s Kappa (0.6511±0.0249 vs. 0.6538±0.0116; t=0.22, p=0.83). None of these differences are statistically significant given the 5-seed sample, and we do not claim KG-DFI’s raw predictive performance exceeds ComplEx-DFI’s, nor the reverse. We nonetheless retain the R-GCN architecture for reasons independent of these metrics. First, the KGE paradigm decouples representation learning—an unsupervised link-prediction objective fit to the full knowledge graph—from the downstream DFI classification objective and requires a separate pre-training stage, whereas KG-DFI’s R-GCN encoder is trained end-to-end jointly with the classification task. Second, R-GCN’s explicit layer-wise message passing supports the multi-hop food–compound–gene–drug path-tracing interpretability analysis in Supplementary File 8; the KGE baselines encode graph structure implicitly in a fixed, pre-trained embedding table and do not offer an analogous mechanism for inspecting intermediate reasoning paths. TransE showed substantially lower performance across all four metrics (F1 = 0.8037, Kappa = 0.5001), consistent with its limited expressiveness for multi-class and asymmetric relation patterns.

**KG-DFI vs. CompGCN**

CompGCN achieved a mean F1 of 0.7890 (±0.0203), substantially below KG-DFI (ΔF1 = −0.0818). CompGCN’s composition-based message passing may be less well-suited to the highly heterogeneous edge type distribution in this KG, which contains 71 unique relation types from diverse sources (FooDB, DRKG, CTD). R-GCN’s relation-specific weight matrices explicitly model each edge type independently, providing greater representational flexibility for heterogeneous graphs.

**GAT fanout sensitivity**

Because GAT requires neighborhood sampling to fit within GPU memory on the full 2.9-million-edge knowledge graph, we first examined how sensitive its performance is to the sampling fanout before settling on the fixed-fanout, 5-seed comparison reported above. Using a single seed (42), GAT was trained at five fanout values (25, 50, 75, 100, and 150 neighbors per node). Results are reported in Table S7.

**Table S7. GAT performance across fanout values (single seed = 42), compared against KG-DFI on the full graph.**

| **Model** | **Graph access** | **F1** | **AUROC** | **AUPRC** | **Kappa** |
| --- | --- | --- | --- | --- | --- |
| KG-DFI (R-GCN) | Full (2.9M edges) | 0.8801 | 0.9163 | 0.9234 | 0.6646 |
| GAT + CrossAttn (fanout=25) | Sampled (~1.0M) | 0.8744 | 0.9430 | 0.9423 | 0.6573 |
| GAT + CrossAttn (fanout=50) | Sampled (~2.1M) | 0.8935 | 0.9403 | 0.9348 | 0.6927 |
| GAT + CrossAttn (fanout=75) | Sampled (~3.1M) | 0.8798 | 0.9311 | 0.9354 | 0.6674 |
| GAT + CrossAttn (fanout=100) | Sampled (~4.2M) | 0.8682 | 0.9387 | 0.9374 | 0.6425 |
| GAT + CrossAttn (fanout=150) | Sampled (~6.3M) | 0.8901 | 0.9469 | 0.9405 | 0.6936 |

Across this range, GAT’s F1 varies non-monotonically between 0.8682 and 0.8935 (range = 0.0253) with no discernible relationship between sampling coverage and performance, consistent with sampling-induced variance rather than a systematic effect of fanout. This instability motivated the controlled, fixed-fanout (fanout = 50), 5-seed comparison reported above (Table S5) as the basis for the primary GAT vs. KG-DFI comparison. Notably, we found that GAT’s results remain somewhat unstable even at this fixed fanout across separately-timed executions of the same code and seeds; see the “Reproducibility note” above for details.
