# Supplementary material for: KG-DFI: A Prediction of Drug–Food Interactions Based on Knowledge Graph Embedding
Source: Comput Struct Biotechnol J. 2026 Aug 18;35(1):0205. doi: 10.34133/csbj.0205 (PMC13481788; doi:10.34133/csbj.0205)
Supplement: Supplementary file 1 [file csbj.0205.f1.zip › Supplementary File 8.docx]

**Supplementary File 8**

**< Multi-hop Path Analysis: Whole-food KG Connectivity >**

This section presents a multi-hop path analysis demonstrating how the KG-DFI knowledge graph encodes biologically meaningful connectivity between food entities and drugs via intermediate food constituents and molecular targets. This analysis addresses the relationship between KG-DFI's whole-food entity representation and the compound-level biological mechanisms that underlie drug–food interactions (DFIs). For each of the four food–drug pairs highlighted in Table 4 of the main text, we trace the representative shortest paths connecting the food entity to the drug entity through the heterogeneous KG.

**Path structure**

All representative paths identified for the Table 4 case-study pairs follow a consistent 3-hop schema:

Food → [CONTAINS] → Compound (food constituent) → [CTD relation] → Gene → [DGIDB relation] → Drug

The first hop traverses FooDB-derived CONTAINS edges, linking the food entity to one of its constituent compounds. The second hop uses a CTD (Comparative Toxicogenomics Database) directed edge encoding a compound–gene biological interaction (e.g., increases_activity, decreases_expression). The third hop traverses a DGIDB edge encoding a gene–drug pharmacological relationship (e.g., ALLOSTERIC MODULATOR, INHIBITOR). This multi-hop structure reveals how a food entity’s constituent compounds serve as mechanistic intermediaries connecting the whole-food entity to specific drug targets.

**Representative paths for Table 4 pairs**

**Figure S5.** Multi-hop knowledge graph paths connecting food entities to drugs for the four case-study pairs in Table 4. Each path follows the schema: Food → [CONTAINS] → Compound → [CTD] → Gene → [DGIDB] → Drug (3 hops). Food nodes (green), food constituent compounds (blue), gene intermediaries (orange), and drug nodes (pink) are shown. Edge labels indicate the KG relation type.

**Table S8.** Representative 3-hop paths in the KG for Table 4 case-study food–drug pairs. Each row describes one representative path

| **Food** | **Food Compound** | **CTD Relation** | **Gene** | **DGIDB Relation** | **Drug** |
| --- | --- | --- | --- | --- | --- |
| Alcoholic beverages (FOOD00854) | CHEMBL3185688 | increases_activity | GABRA1 (Gene::2554) | ALLOSTERIC MODULATOR | Zolpidem (DB00425) |
| Alcoholic beverages (FOOD00854) | CHEMBL190927 | decreases_reaction | IL6 (Gene::3569) | OTHER | Metronidazole (DB00916) |
| Grapefruit (FOOD00256) | CHEMBL1527083 | decreases_expression | Gene::174 | OTHER | Etoposide (DB00773) |
| Grapefruit (FOOD00256) | CHEMBL3184306 | increases_reaction | Gene::84649 | INHIBITOR | Nicotinamide (DB00627) |

**Biological interpretation of representative paths**

Alcoholic beverages (FOOD00854) × Zolpidem. A constituent compound of alcoholic beverages (CHEMBL3185688) increases the activity of GABRA1 (GABA-A receptor α1 subunit, Gene::2554) via a CTD-annotated relationship. GABRA1 is an allosteric modulator target of Zolpidem (DB00425), a sedative-hypnotic drug. This path reflects the well-documented pharmacodynamic synergy between alcohol consumption and benzodiazepine-class sedatives: both enhance GABA-A receptor activity, resulting in additive CNS depression. The KG encodes this clinically meaningful interaction through a coherent 3-hop chain.

Alcoholic beverages (FOOD00854) × Metronidazole. A second alcohol constituent (CHEMBL190927) decreases the reaction of IL6 (Interleukin-6, Gene::3569), which in turn shows an OTHER interaction with Metronidazole (DB00916) in DGIDB. This path is consistent with the known disulfiram-like reaction between alcohol and Metronidazole, in which inflammatory signaling pathways (including IL-6-mediated pathways) are implicated in the adverse reaction.

Grapefruit (FOOD00256) × Etoposide. A grapefruit constituent (CHEMBL1527083) decreases the expression of Gene::174 via CTD. Gene::174 shows an OTHER interaction with Etoposide (DB00773) in DGIDB. Grapefruit’s inhibitory effects on drug-metabolizing enzymes are well characterized, and this path reflects CYP-mediated or related enzyme modulation pathways that affect Etoposide bioavailability.

Grapefruit (FOOD00256) × Nicotinamide. A grapefruit constituent (CHEMBL3184306) increases the reaction of Gene::84649 via CTD. Gene::84649 is identified as an INHIBITOR target of Nicotinamide (DB00627) in DGIDB. This path reflects metabolic interactions involving nicotinamide-related NAD biosynthesis pathways that may be modulated by grapefruit phytochemicals.

**Whole-food entity representation and multi-hop reasoning**

KG-DFI represents each food as a single entity node whose embedding is learned by aggregating information from all neighboring nodes via R-GCN message passing. Unlike compound-level prediction methods, which require a specific constituent compound as input, the food entity embedding implicitly encodes information from all food–compound CONTAINS edges simultaneously. The representative paths above show that the KG encodes biologically relevant food–compound–gene–drug connectivity for the Table 4 pairs. Through R-GCN neighborhood aggregation, the food entity’s embedding accumulates signal from multiple constituent compounds and their associated gene interaction profiles, enabling the model to predict food-level DFIs without requiring explicit compound decomposition at inference time.
