# Supplementary material for: KG-DFI: A Prediction of Drug–Food Interactions Based on Knowledge Graph Embedding
Source: Comput Struct Biotechnol J. 2026 Aug 18;35(1):0205. doi: 10.34133/csbj.0205 (PMC13481788; doi:10.34133/csbj.0205)
Supplement: Supplementary file 1 [file csbj.0205.f1.zip › Supplementary File 9.docx]

**Supplementary File 9**

**< Directional Misclassification Analysis: Knowledge Graph Connectivity and Table 4 Case Studies >**

This section presents an empirical investigation into why some of KG-DFI's high-scoring predictions in Table 4 of the main text correctly detect the presence of a drug–food interaction but misclassify its direction (e.g., predicting Positive when the interaction is reported as Negative or Possible). We examine whether a drug's connectivity within the knowledge graph is associated with this failure mode, using the directional errors observed in Table 4 as a case study.

**Case study: ferric maltol and Table 4's directional errors**

Ferric maltol appears in five of Table 4's twenty drug–food pairs (paired with grapefruit, mung bean, bilberry, oxheart cabbage, and silver linden), and is predicted Positive in all five cases; four of the five are reported as Negative or Possible in the cited literature. To investigate a drug-side explanation for this concentration of errors, we compared ferric maltol's connectivity within the knowledge graph to that of two other iron-based drugs in the same table, iron sucrose and ferrous fumarate, both of which are correctly classified when paired with grapefruit.

**Table S9. Knowledge graph connectivity comparison for ferric maltol and comparator iron-based drugs.**

| **Drug** | **Total degree** | **Percentile (of 925)** | **Drug–drug interaction edges** | **Other relation types (count)** |
| --- | --- | --- | --- | --- |
| Ferric maltol | 9 | 4.0th | 0 | ATC classification (5); disease-related (4) |
| Iron sucrose | 133 | 10.3rd | 123 | ATC classification (5); gene target/carrier (2); drug–gene (2); disease-related (1) |
| Ferrous fumarate | 141 | 10.6th | 120 | Gene target (13); ATC classification (5); disease-related (3) |

Total degree is computed as the number of knowledge graph triples (baseline_kg_enhanced.tsv) in which the drug entity appears as either the head or tail node. Percentile is computed relative to the degree distribution of all 925 DrugBank-mapped drugs in the external validation set described in Section 2.4 of the main text (mean = 1,170, median = 1,092, range = 1–14,731).

Ferric maltol's degree (9) is roughly 15-fold lower than either comparator and places it in the 4th percentile of connectivity among all 925 drugs — markedly sparser even than iron sucrose and ferrous fumarate, which are themselves only moderately connected (10th–11th percentile). The composition of these connections differs qualitatively as well: ferric maltol's nine edges are limited to generic ATC drug-classification links and disease-indication links, with zero DRUGBANK::ddi-interactor-in edges (records of documented interactions with other compounds). By contrast, iron sucrose and ferrous fumarate each have more than 120 such interaction edges. Because R-GCN message passing aggregates information from a node's neighborhood, ferric maltol's embedding is built almost entirely from coarse, category-level signal (“this is an iron supplement”) rather than from any drug-specific interaction history, which plausibly limits the model's ability to distinguish ferric maltol's actual food-interaction profile from a generic iron-supplement pattern. This is consistent with ferric maltol's comparatively recent FDA approval (2019) relative to iron sucrose and ferrous fumarate, and the correspondingly smaller body of accumulated interaction documentation in DrugBank, CTD, and GNBR.

This comparison could in principle be confounded by differences in direct task supervision: if ferric maltol simply appeared in fewer labeled drug–food interaction pairs during training, its poorer performance might reflect a lack of task-specific supervision rather than knowledge graph structure per se. We checked this directly using the 2,822-pair DDID training set: ferric maltol and iron sucrose both appear in zero training pairs, yet iron sucrose is correctly classified in Table 4 while ferric maltol is not. Because these two drugs are equally absent from direct task supervision, this comparison isolates knowledge graph connectivity, rather than training exposure, as the distinguishing factor between them. Ferrous fumarate, the second correctly-classified comparator, does appear in 12 training pairs (19 across the combined train/validation/test split), so its correct classification cannot be as cleanly attributed to connectivity alone; we note this as a partial limitation of the three-drug comparison, though the ferric maltol–iron sucrose pair alone is sufficient to rule out training exposure as the explanation for the observed difference between them.

**Directional error patterns across Table 4**

To assess whether this connectivity-based explanation generalizes beyond the single ferric maltol case, we examined all fifteen Table 4 rows for which a ground-truth literature label is available (five rows report no corroborating literature and are excluded).

**Table S10. Drug knowledge graph degree and directional prediction outcome for all Table 4 pairs with a reported ground-truth label.**

| **Drug** | **Food** | **Degree** | **Predicted** | **Reported** | **Outcome** |
| --- | --- | --- | --- | --- | --- |
| Ferric maltol | Grapefruit | 9 | Positive | Negative | Overestimate |
| Nicotinamide | Alcoholic beverages | 144 | Negative | Possible | Underestimate |
| Ferric maltol | Mung bean | 9 | Positive | Negative | Overestimate |
| Ferric maltol | Bilberry | 9 | Positive | Negative | Overestimate |
| Etoposide | Grapefruit | 1,857 | Negative | Negative | Correct |
| Bictegravir | Grapefruit | 99 | Positive | Positive | Correct |
| Zolpidem | Alcoholic beverages | 1,849 | Negative | Negative | Correct |
| Metronidazole | Alcoholic beverages | 1,444 | Possible | Harmful | Underestimate |
| Iron sucrose | Grapefruit | 133 | Positive | Positive | Correct |
| Ferrous fumarate | Grapefruit | 141 | Positive | Positive | Correct |
| Ferric maltol | Oxheart cabbage | 9 | Positive | Possible | Overestimate |
| Sodium bicarbonate | Alcoholic beverages | 456 | Possible | Possible | Correct |
| Ferric maltol | Silver linden | 9 | Positive | Negative | Overestimate |
| Gefitinib | Grapefruit | 1,368 | Positive | Positive | Correct |
| Upadacitinib | Grapefruit | 61 | Positive | Positive | Correct |

Eight of the fifteen rows (53%) are correctly classified and seven (47%) show a directional error. However, a naive comparison of mean degree between the correct group (745.5) and the incorrect group (233.3 at the row level) is confounded by pseudo-replication: ferric maltol alone accounts for five of the seven incorrect rows, since the same drug is paired with five different foods and its degree (9) is by definition identical across all five. At the drug level — counting each of the three unique drugs involved in an error only once — the incorrect group's mean degree rises to 532.3 (ferric maltol = 9, nicotinamide = 144, metronidazole = 1,444), narrowing the apparent gap with the correct group's 745.5 considerably. Metronidazole in particular is a direct counterexample to a naive “low degree predicts error” hypothesis: its degree (1,444) exceeds that of both iron sucrose and ferrous fumarate, the two correctly classified comparator drugs, yet its prediction is still directionally wrong.

A more precise pattern emerges when errors are split by direction. Predictions can be wrong by overestimating interaction risk (predicting a more severe or positive interaction than reported) or by underestimating it (predicting a less severe or negative interaction than reported). All five overestimation errors in Table 4 involve ferric maltol, the single most sparsely connected drug in the table. Both underestimation errors (nicotinamide, degree = 144; metronidazole, degree = 1,444) involve drugs of moderate-to-high connectivity, and we observe no overestimation error among any well-connected drug in the table. This asymmetry suggests that sparse knowledge graph connectivity — and specifically the absence of drug–drug interaction edges — is most plausibly associated with a specific failure mode, overestimation of interaction risk, rather than with directional misclassification in general. We emphasize that this pattern is grounded in a single drug case (ferric maltol) within a 20-row case study, together with the absence of a counterexample in the same direction, rather than in a statistically validated finding; confirming it would require directional error analysis across a substantially larger and more systematically sampled set of drug–food pairs, which we identify as a direction for future work.

**Relationship to whole-food knowledge graph connectivity (Supplementary File 8)**

Supplementary File 8 demonstrates that rich multi-hop connectivity through food–compound–gene–drug paths enables KG-DFI to correctly detect non-obvious food–drug interactions. The analysis presented here is complementary: it suggests that, conversely, unusually sparse connectivity for a given drug is associated with a specific, identifiable kind of directional error. Together, these two analyses point to knowledge graph connectivity as a first-order determinant of prediction reliability — rich, mechanistically specific connectivity supports accurate detection, while sparse, largely categorical connectivity is associated with overestimation of interaction risk. This motivates connectivity-aware confidence estimation (flagging predictions involving drugs with unusually sparse knowledge graph representation as lower-confidence) as a direction for future work, as noted in the Discussion of the main text.
